# Antiviral activity of recombinant ankyrin targeted to the capsid domain of HIV-1 Gag polyprotein

**DOI:** 10.1186/1742-4690-9-17

**Published:** 2012-02-20

**Authors:** Sawitree Nangola, Agathe Urvoas, Marie Valerio-Lepiniec, Wannisa Khamaikawin, Supachai Sakkhachornphop, Saw-See Hong, Pierre Boulanger, Philippe Minard, Chatchai Tayapiwatana

**Affiliations:** 1 Division of Clinical Immunology, Department of Medical Technology, Faculty of Associated Medical Sciences, Chiang Mai University, Chiang Mai, Thailand 50200; 2 Biomedical Technology Research Unit, National Center for GeneticEngineering and Biotechnology, National Science and Technology Development Agency at the Faculty of Associated Medical Sciences, Chiang Mai University, Chiang Mai 50200, Thailand; 3 Institut de Biochimie et de Biophysique Moléculaire et Cellulaire (IBBMC) UMR-8619, Université de Paris-Sud et CNRS, Orsay Cedex 91405, France; 4 University Lyon 1, 50, avenue Tony Garnier, 69366 Lyon Cedex 07, France; 5 INRA UMR-754, Retrovirus and Comparative Pathology, 50, avenue Tony Garnier, 69366 Lyon Cedex 07, France

**Keywords:** HIV-1, HIV-1 assembly, Gag polyprotein, CA domain, virus assembly inhibitor, ankyrins, artificial ankyrin library, intracellular antiviral agent

## Abstract

**Background:**

Ankyrins are cellular mediators of a number of essential protein-protein interactions. Unlike intrabodies, ankyrins are composed of highly structured repeat modules characterized by disulfide bridge-independent folding. Artificial ankyrin molecules, designed to target viral components, might act as intracellular antiviral agents and contribute to the cellular immunity against viral pathogens such as HIV-1.

**Results:**

A phage-displayed library of artificial ankyrins was constructed, and screened on a polyprotein made of the fused matrix and capsid domains (MA-CA) of the HIV-1 Gag precursor. An ankyrin with three modules named Ank^GAG^1D4 (16.5 kDa) was isolated. Ank^GAG^1D4 and MA-CA formed a protein complex with a stoichiometry of 1:1 and a dissociation constant of *K*_d _~ 1 μM, and the Ank^GAG^1D4 binding site was mapped to the N-terminal domain of the CA, within residues 1-110. HIV-1 production in SupT1 cells stably expressing Ank^GAG^1D4 in both N-myristoylated and non-N-myristoylated versions was significantly reduced compared to control cells. Ank^GAG^1D4 expression also reduced the production of MLV, a phylogenetically distant retrovirus. The Ank^GAG^1D4-mediated antiviral effect on HIV-1 was found to occur at post-integration steps, but did not involve the Gag precursor processing or cellular trafficking. Our data suggested that the lower HIV-1 progeny yields resulted from the negative interference of Ank^GAG^1D4-CA with the Gag assembly and budding pathway.

**Conclusions:**

The resistance of Ank^GAG^1D4-expressing cells to HIV-1 suggested that the CA-targeted ankyrin Ank^GAG^1D4 could serve as a protein platform for the design of a novel class of intracellular inhibitors of HIV-1 assembly based on ankyrin-repeat modules.

## Background

In recent years, significant progress has been made in the control of HIV-1 infections using highly active antiretroviral therapy (HAART). Nevertheless, the occurrence of multi-drug resistant mutants and the side effects of HAART justify the exploration of alternative therapeutic approaches, such as gene therapy [1-5]. Several strategies for anti-HIV gene therapy are currently under development, and certain ones have been tested in hematopoietic cells [6-8]. They can be classified into two major categories: (i) RNA-based agents including antisense, ribozymes, aptamers and RNA interference [9]; (ii) protein-based agents including dominant-negative mutant proteins, intrabodies, intrakines, fusion inhibitors and zinc-finger nucleases [10,11]. The most commonly transduced genes with antiviral potential consist of those encoding derivatives of immunoglobulins. However, the complex structure of these molecules limits their antiviral function within cells, since their stability relies on disulfide bond(s) which rarely occur(s) in the reducing conditions of the intracellular milieu [12-16].

Several methods and novel molecules have been developed to overcome the limitations of antibodies and their derivatives (e.g. scFv), in terms of stability, facility of modifications, robustness, and cost-efficient production [13,17-19]. This is the case for molecules based on protein frameworks or scaffolds which interact with potential therapeutic targets by mimicking the binding process of immunoglobulins to their specific antigens. The ankyrin-repeat proteins represent an attractive scaffold to generate this type of specific binders [20,21]. Analysis of the protein sequence-structure relationship in natural ankyrins has defined consensus ankyrin motifs (or modules), and the results have been used to generate large libraries of artificial proteins, called 'Designed Ankyrin-Repeat Proteins' or DARPins. Several DARPins with desired binding specificity to various target molecules have successfully been isolated from such libraries [12,21-27], including competitors of HIV-1 binding to the viral receptor CD4 [28].

Ankyrins mediate many important protein-protein interactions in virtually all species and are found in all cellular compartments, indicating that these proteins can be adapted to function in a variety of environments, intracellular as well as extracellular [12,20,21,23,25,29,30]. For example, lentiviral vectors pseudotyped with HER2/neu-specific DARPins have been found to efficiently transduce their specific targets, HER2/neu-positive cells [31]. The major advantages of ankyrin-repeat proteins reside in (i) their binding specificity and affinity, as observed in DARPins selected from large libraries; (ii) their solubility and stability, even in the reducing conditions of the intracellular milieu; (iii) their sequence features present in DARPins, which are naturally expressed in human cells: as a consequence, ankyrin-repeat proteins are expected not to be as immunogenic as foreign proteins. Artificial ankyrins are therefore promising candidates as protein interfering reagents capable of acting both extra- and intra- cellularly [24].

In the present study, we designed artificial ankyrin molecules targeted to the HIV-1 Gag polyprotein and evaluated their potential as intracellular therapeutic agents which would negatively interfere with HIV-1 replication, and more specifically with the virus particle assembly machinery. To this aim, we constructed a library of ankyrin-repeat protein library expressed at the surface of recombinant filamentous bacteriophages. This phage-displayed library was screened on immobilized matrix (MA)-capsid (CA) domain (MA-CA) of the HIV-1 Gag precursor (Pr55Gag, or more simply Gag), and a panel of Gag-specific artificial ankyrins were isolated. One particular Gag binder, Ank^GAG^1D4, was selected for further characterization. Ank^GAG^1D4 binding site was mapped to the N-terminal domain of the CA, and SupT1 cells that stably expressed Ank^GAG^1D4 showed a reduced permissiveness to HIV-1 infection. The Ank^GAG^1D4-mediated antiviral effect was found to occur at post-integration steps of the HIV-1 life cycle involving the Gag protein assembly and budding machinery. This study demonstrated the potential of ankyrin-repeat proteins as a novel class of intracellular antivirals and suggested that Ank^GAG^1D4 could serve as a protein platform for the design of efficient intracellular inhibitors of HIV-1 assembly.

## Results

### Design of artificial ankyrin-repeat motifs and construction of an ankyrin library

**Figure 1:**
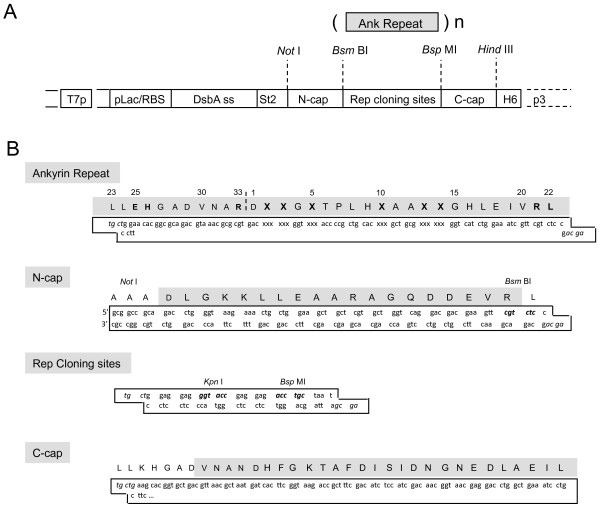
**Schematic construction of the ankyrin repeat library using the pHDiEx acceptor vector**. **(A)**, The mono-ankyrin microgenes were polymerized by insertion/ligation to pH. DiEx double digested by *Bsm *BI and *Kpn *I. The construction was subsequently digested by *Bsp *MI and recircularised by intramolecular ligation. This resulted in the substitution of the region containing the Rep cloning sites by the ankyrin repeats. The number of repeats differed from one clone to another, and usually ranged from 2 to 6 repeats. **(B)**, Detailed sequences of the different DNA regions. Abbreviations: T7p, T7 promoter; pLac/RBS, lactose promoter and Ribosome binding site; DsbA ss, DsbA signal sequence; St2, Strep-Tag2 tag; H6, hexa-histidine tag.

**Figure 2:**
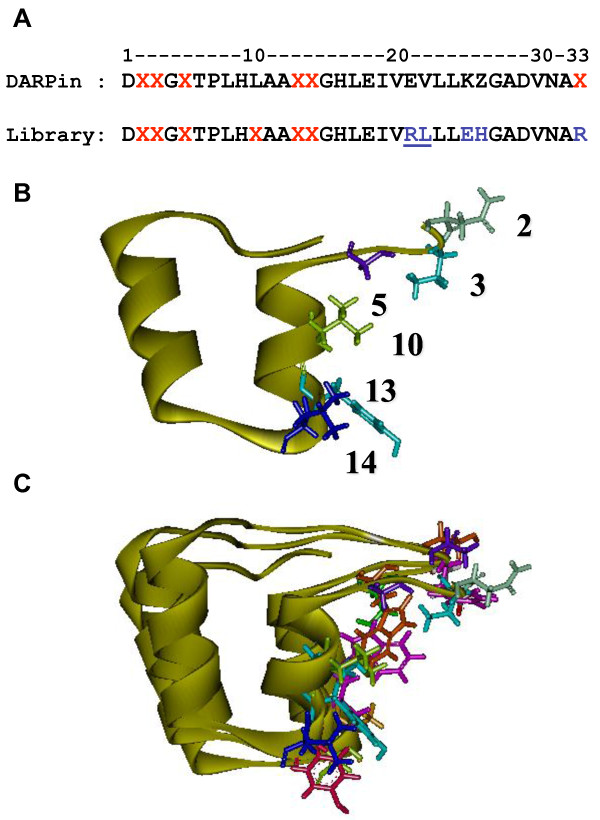
**Consensus sequence and three-dimensional model of ankyrin module**. **(A)**, Sequence comparison between the consensus DARPin repeat motif and the repeat motif of our ankyrin library. The red letters refer to the positions of random amino acids, and blue letters represent the residues which differ from the consensus DARPin sequence. The position of the recognition site for the restriction nuclease *Bsm *BI is underlined. **(B)**, Structural model of one single ankyrin repeat motif (or module). (**C**), Spatial arrangement of three modules belonging to the same ankyrin linear sequence (triple-repeat motif ankyrin molecule). The fixed structure of the repeat motif is presented as a yellow ribbon. The variable amino acids on the solvent-exposed surface are shown as stick pattern; their respective number in the linear sequence is indicated in panel B.

The construction of an artificial ankyrin library implies the randomization of amino acid residues localized on the accessible surface of ankyrin which has a potential interaction with the desired target, while maintaining intact the conserved residues of the consensus repeat modules which form the rigid framework of ankyrin. The consensus sequence of the ankyrin-repeat modules generated in this study was based on previous DARPins libraries [12-14,23,29,30,32], with minor modifications, as described in the Materials and Methods section. For example, the lysine residue (K) at position 25 was substituted for glutamate (E) to prevent a possible repulsion with the positively charged arginine (R) at position 21 (Tables 1 and 2). Our ankyrin library was generated by randomization of amino acids at positions 2, 3, 5, 10, 13, and 14 (Figure 1 and 2). The amino acid side chains at these positions were all oriented outwards and belonged to the same surface-exposed surface of the ankyrin-repeat module (Figure 2B).

**Table 1:** Randomization schemes used to introduce variability at specific positions of ankyrin repeats ^(a)^

Repeat position	Degenerated codons	Subset of encoded amino acid
2	VDK, DMY, RAA	A, D, E, G, H, I, K, L, M, N, Q, R, S, T, V, Y
3	VDK, DMY, VAN	A, D, E, G, H, I, K, L, M, N, Q, R, S, T, V, Y
5	VDK, DMY, VAN, TGG	A, D, E, G, H, I, K, L, M, N, Q, R, S, T, V, W, Y
10	CTG, TGG, TAC, RTC	I, L, V, W, Y
13	KCK, TAC, CGY, VAR	A, E, F, I, K, L, M, Q, R, S, Y
14	KCK, VAR, AAC, SGY, YAY, NTG	A, E, G, H, K, L, M, N, Q, R, S, V, Y

^(a) ^For each position indicated on the leftmost column, the degenerated codons used for the microgene synthesis and the corresponding encoded amino-acids are indicated in the middle and rightmost columns, respectively. Standard letter for mixed bases refer to: N = A/T/G/C, V = A/C/G, D = A/G/T, K = G/T, M = A/C, Y = C/T, R = A/G.

**Table 2:** Amino acid and nucleotides sequences of the ankyrin-repeat microgenes

Repeat position	1 5 10 15 20 25 30
A	D × X G × T P L H L A A × X G H L E I E V L L L K × G A D V N A X
B	D × X G × T P L H **X **A A × X G H L E I **V R **L L L **E H **G A D V N A **R**
C	gac xxx xxx ggt xxx acc ccg ctg cac xxx gct gcg xxx xxx ggt cat ctg gaa atc gtt cgt ctc ctg ctg gaa cac ggc gca gac gta aac gcg cgt
D*	vdk vdk vdk ctg kck kck
	dmy dmy dmy tgg tac var
	raa van van tac cgy aac
	tgg rtc var sgy
	yay
	ntg
E	------------Va --------→ ←----Vb----→ ←----------- Vc ------------→ ←----------------------- C1 ---------------------→ ←-Va-------
	--C3- > ←-------- Vb rev ------→ ←--------------- C2 ------------→ ←---------------- C3 ----------------------

(A), Amino-acid sequence of the previously described ankyrin-repeat module [23].

(B), Amino-acid sequence of the ankyrin-repeat used in the present study.

(C), Nucleotide sequence of the ankyrin microgenes.

(D), Set of partially randomized codons used at each variegated position is indicated. (*), the full sequence of the set of oligonucleotides will be communicated upon request.

(E), Position of the different types of oligonucleotides used to synthetized the ankyrins microgenes along the nucleotide sequence.

The artificial ankyrin-repeat proteins obtained were made of a variable number of ankyrin modules flanked by N- and C-capping sequences (N-cap and C-cap; Figure 1B). The library was generated using the directional polymerization of one ankyrin microgene, each microgene corresponding to one single repeat motif. Polymerization was realized directly into a phagemid vector [33]. This resulted in proteins with variable numbers of repeats and sequence lengths. The length distribution in the library was determined by digesting the phagemid pool with restriction enzymes whose sites were located on both sides of the ankyrin coding sequence, followed by analysis of the DNA fragments by gel electrophoresis. A maximum number of 15 ankyrin repeats was obtained, and the most frequent numbers ranged from 1 to 6.

Our final phage-displayed ankyrin library represented as many as 1.9 × 10^8 ^independent clones. The quality of our library was first evaluated by sequencing proteins from randomly picked clones. Nine out of fifteen clones (60%) presented discontinuous sequences, with sequence frameshifts and stop codons, which likely resulted from errors in oligonucleotide synthesis and/or assembly. Discontinuous sequences occurred with a higher frequency in ankyrins with numerous modules, while most ankyrin molecules with fewer repeats showed correct, open reading frames. The proportion of clones in our library with readthrough ankyrin sequences was also evaluated from the proportion of colonies which expressed C-terminally His-tagged soluble ankyrin protein: 34% (24 out of 72 clones) were found to be positive for the C-terminal His-tag, as monitored by colony filtration blotting (COFI blot; data not shown). We therefore estimated the real diversity of our library to one third of the total number of independent clones, i.e. 6 × 10^7 ^independent ankyrin coding sequences.

### Production and purification of the viral protein target: HIV-1 Gag precursor H_6_MA-CA

The viral target used for screening our phage-displayed ankyrins consisted of the His-tagged recombinant polyprotein H_6_MA-CA, corresponding to the MAp17 and CAp24 domains of the HIV-1 Gag precursor. The rationale for screening our ankyrin library on the MA-CA target was not only to search for MA- or/and CA-binders, but also for ankyrin(s) which recognize(s) the MAp17-CAp24 hinge region, which contains the cleavage site of the viral protease (PR). His-tagged recombinant protein H_6_CA, which corresponded to the mature capsid protein CAp24, was used to identify the structural domains of the Gag precursor which contained the ankyrin-binding site. Large amounts of recombinant H_6_MA-CA and H_6_CA proteins were produced in Sf9 cells infected with recombinant baculoviruses BV-H_6_MA-CA or BV-H_6_CA, and the recombinant Gag proteins purified by affinity chromatography on nickel-sepharose column.

### Screening of Gag-binding ankyrins using the phage-display method

The phage-displayed library of ankyrins was amplified using a conventional protocol [34,35], and Gag-binders were isolated by three rounds of selection/elution from surface-immobilized H_6_MA-CA protein. Elution of H_6_MA-CA-bound phages was performed using acidic buffer for the first two rounds, followed by specific ligand elution using excess of soluble H_6_MA-CA protein as the competititor in the third round [34,35]. Phage clones were picked at random in each eluate, and tested by ELISA for binding to H_6_MA-CA. Only 20% of Gag-binders were found in the first eluate, whereas a significant enrichment was observed in the second and third eluates, with 70% of Gag-binders in both. Clones which gave a signal 5-fold over the background signal were picked in all eluates and sequenced. All positive clones showed two or three ankyrin repeats flanked by N-cap and C-cap. Three different clones, referred to as Ank^GAG^1B8, Ank^GAG^1D4 and Ank^GAG^6B4 and containing three ankyrin modules each, were identified several times; they were therefore selected for further studies.

To evaluate the specificity of our Gag-binders, an irrelevant target protein, αRep-A3, was used in lieu of H_6_MA-CA. Protein αRep-A3, previously described under the acronym αRep-n4-a in our previous study [33], is an artificial alpha-helicoidal repeat protein (αRep) based on thermostable HEAT-like repeats, which folds cooperatively and shows a high stability [33]. Our phage-displayed ankyrin library was screened on immobilized αRep-A3 protein, and αRep-A3-bound clones were checked for binding specificity and sequenced. One ankyrin clone with a high affinity and specificity for the αRep-A3 target, referred to as Ank^A3^2D3, was used as the irrelevant control of H_6_MA-CA binders in the rest of the present study.

### Gag-ankyrin interaction

Gag- and αRep-A3-binding ankyrins were purified, chemically biotinylated, and assayed for their capacity of binding to their specific target *in vitro*. Importantly, no change was detected in the interaction of the three Gag-binders Ank^GAG^1B8, Ank^GAG^1D4 and Ank^GAG^6B4, and of control αRep-A3-binder Ank^A3^2D3, with their respective substrates, as determined by ELISA (data not shown). This indicated that biotinylation did not alter their Gag- or αRep-A3-specific binding activity.

**Figure 3:**
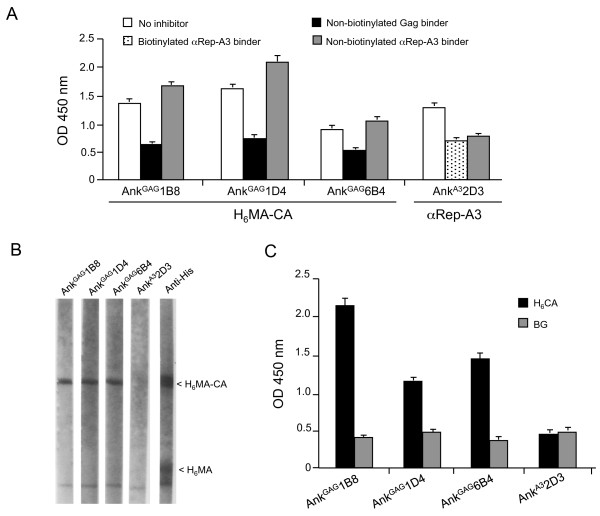
**Gag-binding activity of artificial ankyrins**. **(A)**, ***Competition ELISA***. Samples of biotinylated Gag-binders Ank^GAG^1B8, Ank^GAG^1D4 and Ank^GAG^6B4, and of control biotinylated αRep-A3-binder Ank^A3^2D3 were mixed with their corresponding non-biotinylated form (black bars), or mixed with irrelevant soluble target (grey bars), or mixed with buffer containing no inhibitor (white bars). Mixtures were added to H_6_MA-CA- or αRep-A3-coated wells, as indicated at the bottom of the panel. Bound-ankyrins were detected by addition of HRP-conjugated extravidin, followed by the TMB substrate. **(B)**, ***Far Western blotting***. Lysates of BV-H_6_MA-CA-infected Sf9 cells were electrophoresed in SDS-gel, proteins transferred to a PVDF membrane, and membrane cut into strips. Gag-binding activity was determined by incubation of the strips with the different biotinylated ankyrins Ank^GAG^1B8, Ank^GAG^1D4, Ank^GAG^6B4, and Ank^A3^2D3, as indicated on top of the strips. On the rightmost strip, the respective positions of the Gag proteins H_6_MA-CA and H_6_MA were determined using anti-histidine tag antibody (arrowheads). **(C)**, ***Indirect ELISA***. H_6_CA was captured on nickel-coated plate, and used as substrate for binding assay of biotinylated ankyrins Ank^GAG^1B8, Ank^GAG^1D4, Ank^GAG^6B4, and Ank^A3^2D3. Bound-ankyrins were quantitated as in **(A)**. BG, background signal.

The degree of Gag-specificity of biotinylated Ank^GAG^1B8, Ank^GAG^1D4 and Ank^GAG^6B4 was evaluated in the presence of specific or nonspecific competitors, and tested in ELISA using H_6_MA-CA-coated wells. Controls consisted of Ank^A3^2D3 and αRep-A3-coated wells. Competitors were (i) the same ankyrin protein in its non-biotinylated form and (ii) non-biotinylated αRep-A3 protein. Ank^GAG^1B8, Ank^GAG^1D4, Ank^GAG^6B4 and Ank^A3^2D3 were all competed with their respective non-biotinylated versions, while no significant competition was observed between ankyrins Ank^GAG^1B8, Ank^GAG^1D4, Ank^GAG^6B4 on one hand, and αRep-A3 protein on the other hand (Figure 3A). Interestingly, Ank^GAG^1D4 showed the highest signal of binding to the H_6_MA-CA target, and the highest competition effect was observed with non-biotinylated Ank^GAG^1D4 (Figure 3A).

### Identification of the ankyrin binding domain on HIV-1 Gag precursor

The structural domain of Pr55Gag recognized by each of the three Gag-binders Ank^GAG^1B8, Ank^GAG^1D4 and Ank^GAG^6B4 was determined by Far Western blot analysis and ELISA. Lysates of H_6_MA-CA-expressing Sf9 were analyzed by SDS-PAGE, and proteins transferred to PVDF membranes. Spontaneous cleavage of H_6_MA-CA by insect cell proteases resulted in the occurrence of His-tagged N-terminal domain, H_6_MA, migrating as the mature matrix protein of the virion, MAp17 (Figure 3B; control, rightmost lane). All three Gag-binders, Ank^GAG^1B8, Ank^GAG^1D4 and Ank^GAG^6B4, reacted with H_6_MA-CA on blot, but not with H_6_MA (Figure 3B). This indicated that the ankyrin binding site was not located in the MA domain, but in the CA domain. As expected, no reaction was obtained with the control αRep-A3-binder Ank^A3^2D3 (Figure 3B). The reactivity towards the CA domain was confirmed by indirect ELISA, using recombinant H_6_CA protein immobilized on nickel-coated wells. Positive signals with the CA protein were detected with all three Gag-binders, but not with Ank^A3^2D3 (Figure 3C). This indicated that the binding sites of Ank^GAG^1B8, Ank^GAG^1D4 and Ank^GAG^6B4 on H_6_MA-CA protein were all situated in the CA domain.

### Biochemical characterization of Gag-binding ankyrins

**Figure 4:**
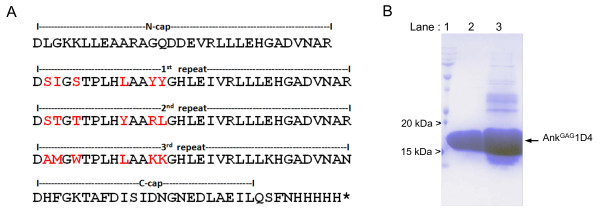
**Characterization of Ank^GAG^1D4**. **(A)**, Amino acid sequence of the Ank^GAG^1D4 protein, represented by using the single letter code. The variable residues at the six predefined positions on the ankyrin solvent-exposed surface were highlighted in red. **(B)**, SDS-PAGE analysis of Ank^GAG^1D4. The Ank^GAG^1D4 protein was first isolated by affinity chromatography on nickel-column (HisTrap column; lane 3), and further purified by gel filtration chromatography (Sephadex G-75 column; lane 2). Lane 1, molecular mass markers.

As shown in Figure 3C, Ank^GAG^1B8 reacted with H_6_CA with the highest apparent affinity. However, DNA sequencing showed several nonconservative amino acid substitutions within the highly structured scaffold domain of the Ank^GAG^1B8 modules, as well as in Ank^GAG^6B4. Since these mutations could adversely affect the ankyrin-repeat motifs, Ank^GAG^1B8 and Ank^GAG^6B4 were excluded from our next analyses, and only Ank^GAG^1D4 was selected for further characterization. DNA sequencing revealed that Ank^GAG^1D4 protein comprised of three ankyrin modules, each containing different types of amino acids at the six assigned positions for variable residues (Figure 4A). The oligo-histidine tag allowed us to purify Ank^GAG^1D4 protein to homogeneity by using a two-step chromatographic procedure, (i) affinity chromatography (Figure 4B, lane 3), and (ii) gel filtration (Figure 4B, lane 2). SDS-PAGE analysis showed that Ank^GAG^1D4 migrated with an apparent molecular mass of 16.5 kDa (Figure 4B), consistent with the theoretical mass 17.9 kDa for a protein of 163 amino acid residues.

### Mapping of the Ank^GAG^1D4 binding site on the CA domain

**Figure 5:**
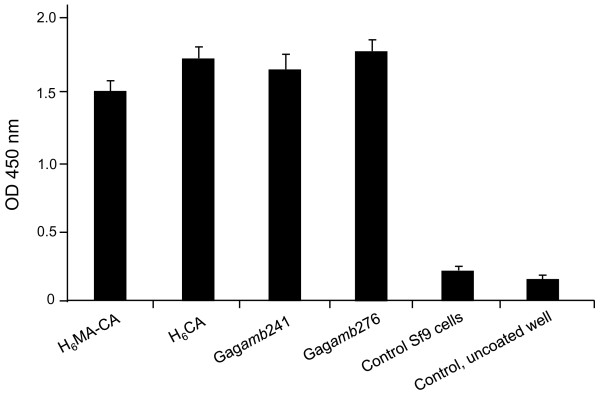
**Mapping of the Ank^GAG^-1D4 binding site on HIV-1 Gag CA domain**. The binding activity of biotinylated Ank^GAG^1D4 protein was tested in ELISA towards surface-immobilized lysates of mock-infected Sf9 cells, or baculovirus-infected Sf9 cells expressing recombinant H_6_MA-CA, H_6_CA, Gag*amb*241, or Gag*amb*276 protein. The quantity of bound Ank^GAG^1D4 was assayed by reaction with streptavidin-HRP. Data presented are from triplicate experiments (m ± SEM).

A more refined mapping of the ankyrin binding site on the CA domain was performed using carboxyterminal deletion mutants of Gag expressed as recombinant proteins in baculovirus-infected cells. Gag*amb*276 and Gag*amb*241 mutants carried an amber stop codon in the Pr55Gag sequence at positions 276 and 241, respectively [36]. Both recombinant Gag proteins had in common the MA domain, plus 110 residues of the CA domain for Gag*amb*241, and 145 residues of the CA domain for Gag*amb*276 [36]. Ank^GAG^1D4 was found to bind to both C-truncated Gag proteins (Figure 5). This restricted the Ank^GAG^1D4 binding site to the N-terminal region of the CA domain spanning residues 1 to 110, corresponding to positions 132-241 in the Pr55Gag sequence of 500 amino acids.

### Gag-binding parameters of Ank^GAG^1D4

**Figure 6:**
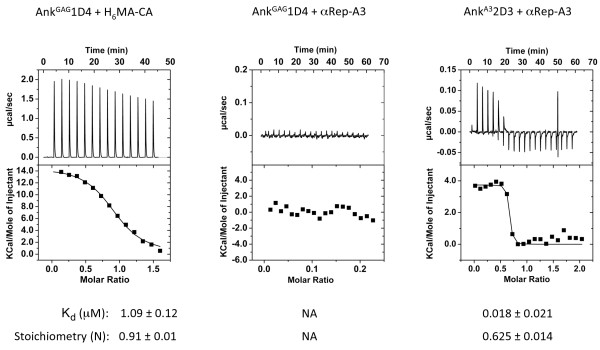
**Gag-binding activity of Ank^GAG^1D4 analyzed by microcalorimetry (ITC): affinity and specificity**. The affinity of Ank^GAG^1D4 towards its Gag substrate H6MA-CA (leftmost panel) and the affinity of Ank^A3^2D3 towards its αRep-A3 substrate (rightmost panel) were determined by ITC measurements of ankyrin/substrate mixtures at various protein ratios. The Gag-binding specificity was evaluated by ITC analysis of the mixture of Ank^GAG^1D4 and irrelevant substrate αRep-A3 at various protein ratios (middle panel). The parameters of each binding reaction, *K*_d _and stoichiometry (N), are shown under the corresponding panel. NA, calculation not applicable.

The specificity and binding parameters of Ank^GAG^1D4 to its H_6_MA-CA substrate were determined by microcalorimetry (ITC). Titration of increasing amounts of Ank^GAG^1D4 protein into sample cell containing purified H_6_MA-CA protein gave the approximate value of 1 μM for the dissociation constant (*K*_d_) of the specific reaction of the binder with its target protein (Figure 6; leftmost top panel). In control experiments, no interaction was detected between Ank^GAG^1D4 and αRep-A3 (Figure 6; middle top panel). By comparison, Ank^A3^2D3 interacted with its substrate αRep-A3 with a *K*_d _= 18 nM (Figure 6; rightmost top panel).

The stoichiometry (N) of the interacting molecules in the protein complexes and the number of binding sites were calculated from the fitting curves of ITC data. The stoichiometry of protein monomers was found to be N = 0.91 for the pair Ank^GAG^1D4/H_6_MA-CA, and N = 0.62 for the control pair Ank^A3^2D3/αRep-A3 (Figure 6). The αRep-A3 protein is known to occur as a homodimer [33], and the experimental value of 0.62 was close to the theoretical ratio of 0.5. The data therefore suggested that one molecule of Ank^A3^2D3 bound to an αRep-A3 homodimer to form a ternary Ank^A3^2D3/(αRep-A3)_2 _complex. By contrast, Ank^GAG^1D4 bound to the H_6_MA-CA monomer in a 1-to-1 binary complex.

### Construction of SupT1 cell lines stably expressing Gag-binding ankyrin proteins

**Figure 7:**
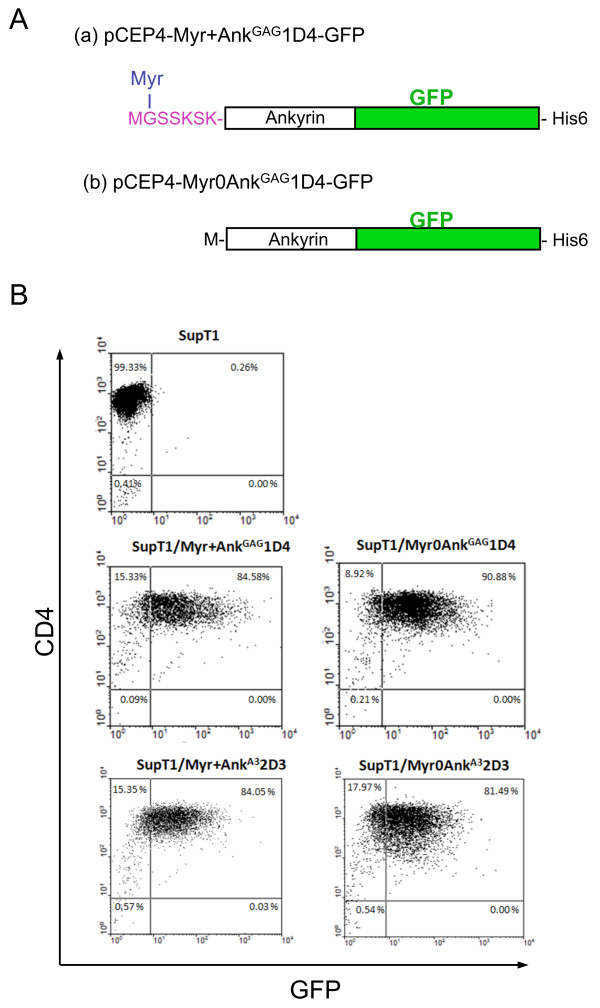
**Characterization of SupT1 cells stably expressing artificial ankyrins**. **(A)**, ***Ankyrin constructs***. Schematic representation of the artificial ankyrin constructs designed for stable expression in SupT1 cells, using pCEP4-based vector. Histidine-tag and green fluorescence protein (GFP; green box) were inserted at the C-terminus and in-phase with the ankyrin sequence (white box). Addition of a N-myristoylation signal (in purple red letters) to the Myr+Ank^GAG^1D4-GFP clone resulted in the removal of the N-terminal methionine (M) and covalent linkage of myristic acid chain (Myr; in blue letters) to glycine-2 (G). **(B)**, ***Flow cytometry***. Expression of CD4 molecules at the surface of control SupT1 cells, SupT1/Myr+Ank^GAG^1D4-GFP and SupT1/Myr0Ank^GAG^1D4-GFP cells. Flow cytometry analysis was performed on nonpermeabilized cells, using monoclonal antibody against CD4, followed by PE-conjugated goat anti-mouse IgG.

Two pCEP4-based episomal plasmids encoding the Ank^GAG^1D4 protein in its Myr+ and Myr0 versions, respectively, and fused to His-tagged GFP at the N-terminus (Figure 7A) were transfected into the SupT1 cell line. Clones that stably expressed Ank^GAG^1D4-GFP protein (SupT1/Myr+Ank^GAG^1D4-GFP and SupT1/Myr0Ank^GAG^1D4-GFP) were identified by fluorescent microscopy, isolated and expanded under the hygromycin-B selection. Two control SupT1 cell lines harboring the pCEP4 plasmids encoding the Myr+ and Myr0 versions of Gag-irrelevant Ank^A3^2D3-GFP (SupT1/Myr+Ank^A3^2D3-GFP and SupT1/Myr0Ank^A3^2D3-GFP) were generated in parallel. Confocal microscopy showed that Myr+Ank^GAG^1D4-GFP and Myr+Ank^A3^2D3-GFP localized in both the cytoplasm and the plasma membrane, as expected for N-myristoylated proteins, whereas Myr0Ank^GAG^1D4-GFP and Myr0Ank^A3^2D3-GFP showed a diffuse cytoplasmic fluorescence. Flow cytometry analysis showed that almost 80% of ankyrin-expressing cells were GFP-positive, and that Myr+Ank^GAG^1D4-GFP or Myr0Ank^GAG^1D4-GFP did not negatively interfere with the surface expression of CD4 (Figure 7B). The status of CD4 molecules, the primary receptors of HIV-1, was important to assess in ankyrin-expressing cells prior to HIV-1 infection, in order to ensure that SupT1/Myr+Ank^GAG^1D4-GFP and SupT1/Myr0Ank^GAG^1D4-GFP cells could serve as host cells for testing the functionality of Ank^GAG^1D4 as antiviral agent.

### HIV-1 infection of ankyrin-expressing SupT1 cell lines

**Figure 8:**
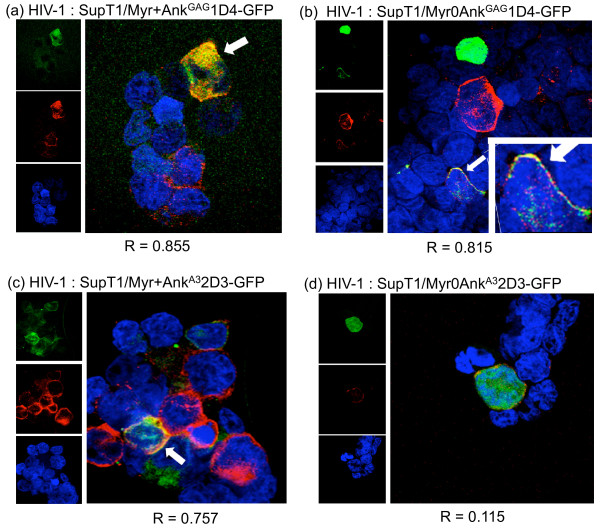
**Confocal microscopy of HIV-1-infected, ankyrin-expressing SupT1 cells**. HIV-1-infected SupT1/Myr+Ank^GAG^1D4-GFP (panel **a**), SupT1/Myr0Ank^GAG^1D4-GFP (**b**), SupT1/Myr+Ank^A3^2D3-GFP (**c**), and SupT1/Myr0Ank^A3^2D3-GFP cells (**d**) were collected at day 11 pi, permeabilized, immunolabeled with anti-CAp24 mAb and PE-conjugated anti-mouse IgG F(ab')_2 _antibody (red signal), and nuclei counter-stained in blue with DAPI. Ankyrins were detected by their GFP-tag. Merged images are enlarged and shown on the right side of each panel. White arrows point to cells showing colocalization of ankyrin and Gag proteins. R, the Pearson correlation coefficient for signal colocalization, was determined using the Olympus FluoView software.

SupT1/Myr+Ank^GAG^1D4-GFP, SupT1/Myr0Ank^GAG^1D4-GFP, SupT1/Myr+Ank^A3^2D3-GFP and SupT1/Myr0Ank^A3^2D3-GFP cells were infected with HIV-1_NL4-3 _virus at MOI 10 for 16 h at 37°C. Cells were harvested at day 11 post-infection (pi), and examined in confocal microscopy after permeabilization and reaction with anti-CAp24 mAb and PE-conjugated anti-mouse IgG-F(ab')_2 _antibody. In infected cells expressing Myr+Ank^GAG^1D4-GFP, the Gag and GFP signals superimposed in both cytoplasmic compartment and at the plasma membrane, as expected for two N-myristoylated, membrane-targeted partner proteins (Figure 8a). Gag and Gag-irrelevant N-myristoylated ankyrin Myr+Ank^A3^2D3-GFP were also both addressed to the plasma membrane, but showed a lesser degree of colocalization (Figure 8c). No significant colocalization was detected for Gag and the non-N-myristoylated, Gag-irrelevant ankyrin Myr0Ank^A3^2D3-GFP (Figure 8d). Interestingly, a significant degree of colocalization was observed for Gag and the non-N-myristoylated Gag-binder Myr0Ank^GAG^1D4-GFP (Figure 8b). This implied that Gag and Myr0Ank^GAG^1D4-GFP proteins interacted within the cytoplasm and were addressed as a complex to the plasma membrane, via the N-myristoylated signal carried by the Gag protein partner.

### Negative effect of Ank^GAG^1D4 on HIV-1 production

**Figure 9:**
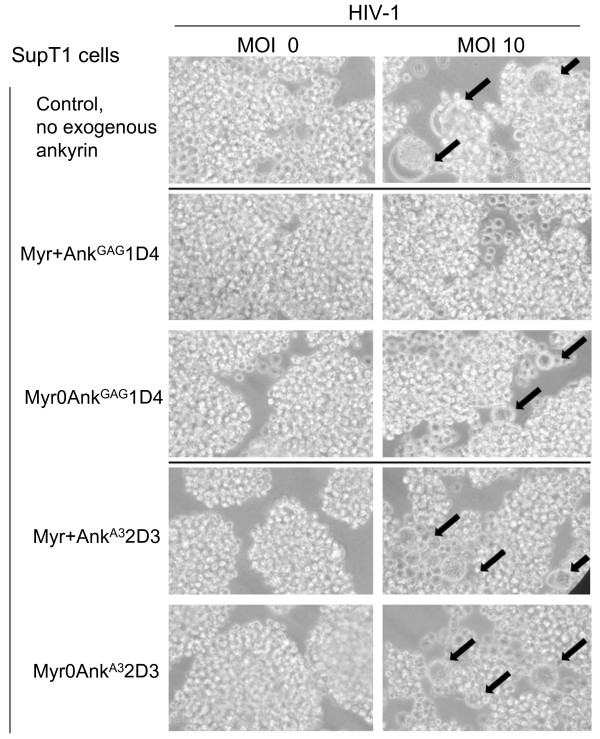
**HIV-1-induced syncytium formation**. SupT1/Myr+Ank^GAG^1D4, SupT1/Myr0Ank^GAG^1D4, SupT1/Myr+Ank^A3^2D3 and SupT1/Myr0Ank^A3^2D3 were mock-infected (MOI 0; left column) or infected with HIV-1 (MOI 10, right column). Cells were observed at 400X magnification using an inverted microscope. Black arrows point to syncytia.

The possible antiviral activity of Ank^GAG^1D4 on HIV-1 assembly and budding was first evaluated by syncytium formation. SupT1 cells expressing Myr+Ank^GAG^1D4-GFP, Myr0Ank^GAG^1D4-GFP, Myr+Ank^A3^2D3-GFP and Myr0Ank^A3^2D3-GFP, respectively, were infected with HIV-1_NL4-3 _virus at MOI 10 for 16 h at 37°C, and examined by phase contrast microscopy at day 11 pi. Numerous syncytia were observed in control samples of HIV-1-infected SupT1 cells (Figure 9, upper row) as well as in HIV-1-infected SupT1/Myr+Ank^A3^2D3 and SupT1/Myr0Ank^A3^2D3 cells (Figure 9, two bottom rows). However, very few syncytia were observed in HIV-1-infected SupT1/Myr0Ank^GAG^1D4 cells (Figure 9, third row from the top), and very rare, if any, in HIV-1-infected SupT1/Myr+Ank^GAG^1D4 cells (Figure 9, second row from the top).

**Figure 10:**
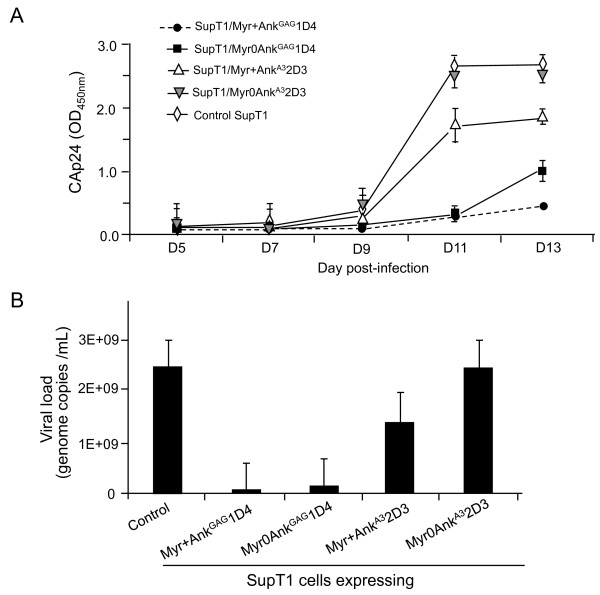
**Ank^GAG^1D4-mediated inhibitory effect on HIV-1 replication**. **(A)**, ***CAp24 titration***. SupT1/Myr+Ank^GAG^1D4 and SupT1/Myr0Ank^GAG^1D4 stably expressed the N-myristoylation and non-N-myristoylation versions of the H6MA-CA-binding ankiryn Ank^GAG^1D4, respectively. SupT1/Myr+Ank^A3^2D3 and SupT1/Myr0Ank^A3^2D3 expressed the N-myristoylation and non-N-myristoylation versions of the αRep-A3-binding ankyrin Ank^A3^2D3, respectively. Cells were infected with HIV-1 _NL4-3 _at MOI 10, and cell culture supernatants collected at time intervals (5, 7, 9, and 11 days pi) and virus progeny titers determined by CAp24 assays, using ELISA. Results shown are mean (m) from triplicate experiments ± SEM. **(B)**, ***Viral load***. The copy numbers of viral genome were determined in the culture supernatants collected on day-11, using Cobas Amplicor tests. Data presented are from triplicate experiments (m ± SEM). The average values were the following: Control SupT1 cells = 2, 470 × 10^7 ^copies/mL; SupT1/Myr+Ank^GAG^1D4 = 4 × 10^7^; SupT1/Myr0Ank^GAG^1D4 = 15 × 10^7 ^; SupT1/Myr+Ank^A3^2D3 = 140 × 10^7 ^; SupT1/Myr0Ank^A3^2D3 = 245 × 10^7^.

Virus yields were quantitated in the extracellular medium of ankyrin-expressing SupT1 cells infected with HIV-1_NL4-3 _virus, in the same conditions as above. Culture supernatants were collected at different times pi, and virus progeny titer indirectly determined using ELISA/CAp24. A significant reduction of extracellular levels of CAp24 was observed at days 11 and 13 pi in the supernatants of SupT1/Myr+Ank^GAG^1D4-GFP and SupT1/Myr0Ank^GAG^1D4-GFP cells, compared to control cells, nontransduced HIV-1-infected SupT1 cells and SupT1 cells expressing the Gag-irrelevant ankyrin Myr+Ank^A3^2D3-GFP (Figure 10A). There was a slight decrease of CAp24 levels in the culture medium of SupT1/Myr+Ank^A3^2D3-GFP, compared to control SupT1 cells, and this effect was less pronounced in SupT1/Myr0Ank^A3^2D3-GFP cells, which expressed a Gag-irrelevant, non-N-myristoylated ankyrin, (Figure 10A). The possibility that the increase in CAp24 yields at day 13 pi might be due to Ank^GAG^1D4-escape HIV-1 mutant(s) was investigated: no mutation in the *gag *gene was found in RT-PCR amplicons derived from the HIV-1 progeny of SupT1/Myr+Ank^GAG^1D4 cells harvested at day 13. However, this did not exclude that Ank^GAG^1D4-resistant *gag *mutants could be found after a higher number of passages. Long-term cultures of HIV-1-infected SupT1 cells stably expressing Ank^GAG^1D4 will be necessary to evaluate the viral genetic barrier to Ank^GAG^1D4.

Viral loads were also determined at day-11 in the extracellular media, and the data confirmed the ELISA/CAp24. A significant inhibitory effect of Myr+Ank^GAG^1D4 on HIV-1 replication was observed, with an average 600-fold lower virus progeny production, compared to control, nontransduced HIV-1-infected SupT1 cells (2, 500 × 10^7 ^genome copies/mL; Figure 10B). A significant decrease in HIV-1 production was also observed with the non-N-myristoylated ankyrin Myr0Ank^GAG^1D4, although to a lesser degree compared to its N-myristoylated version (160-fold less; Figure 10B). These results indicated that the antiviral function of Ank^GAG^1D4 occurred in both compartments, plasma membrane and cytoplasm, but with a higher efficiency when the ankyrin molecules were addressed to the plasma membrane (Figure 10B). This suggested that the antiviral function carried by Ank^GAG^1D4 occurred at late step(s) of the virus life cycle, e.g. the assembly and budding of virus particles. As observed in ELISA/CAp24, SupT1 cells expressing Gag-irrelevant, N-myristoylated ankyrin molecule Myr+Ank^A3^2D3-GFP showed some decrease (17-fold) in virus production (140 × 10^7 ^genome copies/mL; Figure 10B).

### Ank^GAG^1D4-mediated antiviral activity in HIV-1 life cycle: early versus late step(s)

As a partner of the HIV-1 structural protein CAp24, Ank^GAG^1D4 might interfere with various step(s) of the HIV-1 life cycle. This included (i) virus uncoating, (ii) intracellular trafficking of incoming viruses, (iii) nuclear import of the viral preintegration complex, at early times, (iv) Gag oligomerisation, (v) virus particle assembly, and (v) extracellular budding, at late times post infection. To address this issue, integration events were evaluated by PCR amplification of *Alu-gag *junctions in HIV-1-infected, Ank^GAG^1D4-expressing SupT1 cells harvested at day 11 pi. Control samples consisted of HIV-1-infected, nontransfected SupT1 cells and SupT1 cells expressing Gag-irrelevant, Ank^A3^2D3 ankyrin. There was no significant difference between Ank^GAG^1D4-expressing SupT1 cells and control cells (Table 3), suggesting that the Ank^GAG^1D4-mediated antiviral effect took place at the post-integration phase of the virus life cycle. To validate this negative result, control experiments of integration blockage were carried out using the HIV-1 integrase inhibitor Raltegravir™ (RAL). RAL was added at increasing molarities (1, 10 and 100 nM) to the SupT1 cell culture medium 24 h prior to HIV-1 infection, and maintained for 7 days [37]. No significant alteration of the cell viability was observed within this molarity range (Additional File 1). No viral integration was detectable at RAL molarities over 10 nM (Table 4 and Additional File 1), a result which was consistent with the IC_50 _value of 10 nM for RAL [37].

**Table 3:** HIV-1 integration events in control and ankyrin-expressing SupT1 cells ^(a)^

Sequence amplified	No ankyrin	Myr+Ank^GAG^1D4	Myr0Ank^GAG^1D4	Myr+Ank^A3^2D3
Alu-*gag *junctions	28.3 ± 0.2	28.6 ± 0.3	30.5 ± 0.1	27.8 ± 0.2
GAPDH	25.7 ± 0.4	26.1 ± 0.5	25.5 ± 0.5	24.5 ± 0.1

^(a) ^The level of HIV-1 integration in SupT1 cells harvested at day 11 pi was evaluated by quantitative PCR amplification of host cell DNA extracts, using primers specific to *Alu*-*gag *junctions, and to cellular *GAPDH *gene used as the internal control. Figures shown in the Table are *C*ts values, mean ± SD (*n *= 3).

**Table 4:** HIV-1 integration events in control and Raltegravir-treated SupT1 cells ^(a)^

Sequence amplified	Raltegravir (nM)			
	0	1	10	100
Alu-*gag *junctions	26.9 ± 0.2	36.1 ± 0.3	ND	ND

GAPDH	23.3 ± 0.5	23.7 ± 0.4	24.1 ± 0.3	23.9 ± 0.2

^(a) ^Aliquots of HIV-1-infected SupT1 cells were pretreated with Raltegravir at 0, 1, 10, and 100 nM, respectively, for 24 hr prior to HIV-1 infection (MOI 10), and the drug maintained at the indicated concentrations for 7 days. Host cell genomic DNA was extracted on day 7 pi, and the level of HIV-1 integration in SupT1 cell lines was evaluated by quantitative PCR amplification of DNA extracts, using primers specific to *Alu*-*gag *junctions and to cellular *GAPDH *gene as the internal control. Figures shown in the Table are *C*ts values (mean ± SD, *n *= 3). ND, not detectable (below the threshold of detection).

**Figure 11:**
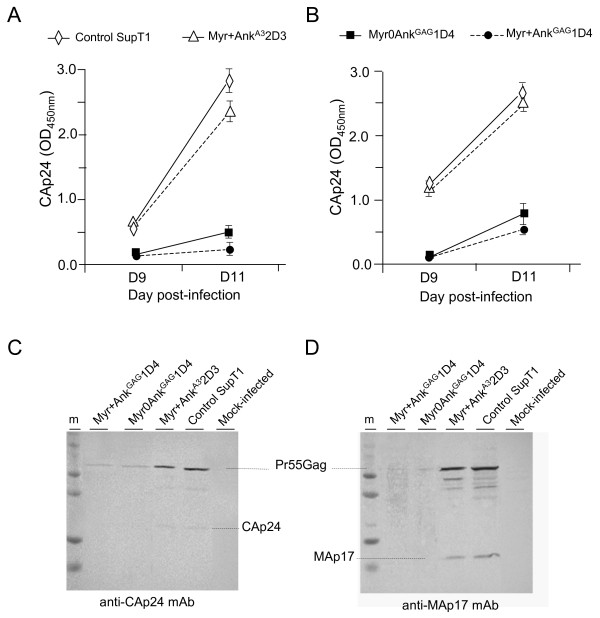
**Effect of ankyrin expression on Gag protein levels in HIV-1-infected SupT1 cells**. Control and ankyrin-expressing SupT1 cell lines were infected with HIV-1_NL4-3 _inoculum at 10 MOI for 16 h at 37°C. After washing with serum-free medium, cells were resuspended in prewarmed medium containing 500 μg/mL hygromycin B and 10% FCS, and seeded into 6-well plates. Cells were harvested and lysed at day 9 and 11 pi. Cell lysates were fractionated into nuclear pellet, membrane compartment and cytosol, and each fraction assayed for CAp24 content by ELISA, and Gag proteins by SDS-PAGE and Western blot (WB) analysis. Protein concentration in all samples was normalized to 10 μg/mL. **(A)**, ELISA of whole cell lysates (WCL); **(B)**, ELISA of membrane/particulate fractions. Data presented are mean from triplicate experiments, with error bars indicating the standard error to mean. **(C, D)**, SDS-PAGE and WB analysis of WCL. Samples were taken at day 11 pi, and analyzed using anti-CAp24 (C) and anti-MAp17 mAb (D).

To further dissect the nature of the post-integration blockage of HIV-1 provoked by Ank^GAG^1D4, the fate of the viral target of Ank^GAG^1D4, the Gag protein, was analyzed in HIV-1-infected SupT1 cells harvested at late times pi and subjected to cell fractionation. Whole cell lysates and cell fractions were assayed for Gag content by ELISA/CAp24, and the Gag protein pattern analysed by SDS-PAGE and Western blotting. The CAp24 levels were significantly lower in Myr+Ank^GAG^1D4- and Myr0Ank^GAG^1D4-expressing cells, compared to control cells expressing no exogenous ankyrin or the Gag-irrelevant ankyrin Ank^A3^2D3 (Figure 11). A similar decrease was observed in the whole cell lysate and membrane fraction (Figure 11, compare panels A and B), implying that the antiviral effect of Ank^GAG^1D4 did not involve the trafficking of Gag to the plasma membrane.

Western blot analysis showed a drastic reduction of all Gag protein species in Myr+Ank^GAG^1D4- and Myr0Ank^GAG^1D4-expressing cells compared to control cells (Figure 11C, D). This pattern excluded a possible interference of Ank^GAG^1D4 with the proteolytic processing of Gag, which might provoke a premature cleavage of the Pr55Gag precursor.

### Viral specificity of Ank^GAG^1D4

**Figure 12:**
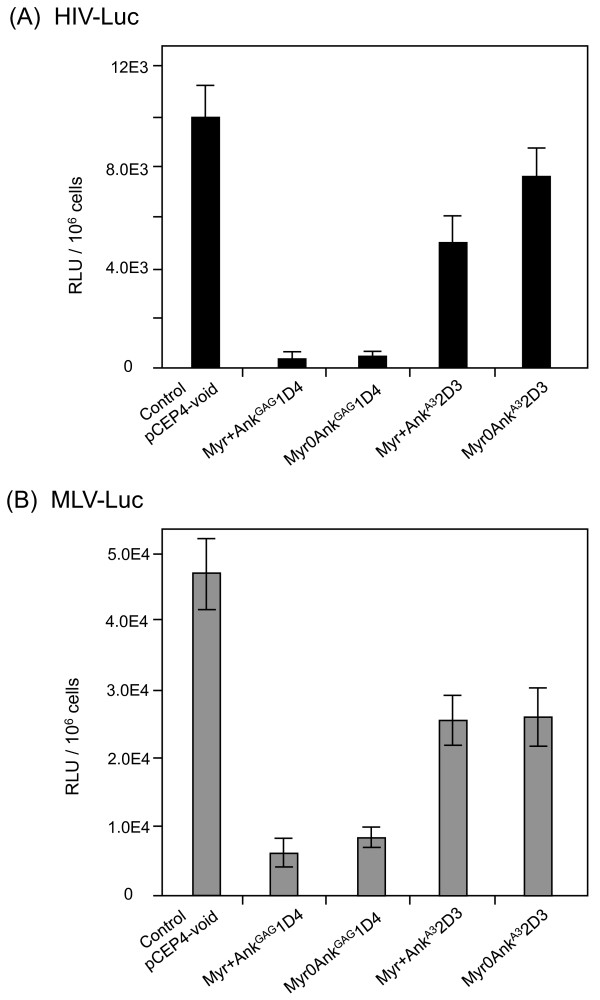
**Influence of Ank^GAG^1D4 on the HIV-Luc **(A) **and MLV-Luc vector (B) yields**. VSV-G-pseudotyped HIV-Luc released by 293T cells cotransfected with pNL4-3Luc(R-E-), phCMV-G and pCEP4-ankyrin **(A)**, and VSV-G-pseudotyped MLV-Luc vector released by GP2-293-Luc cells cotransfected with phCMV-G and pCEP4-ankyrin (**B**), were titrated on 293T cells, using the luciferase assay. RLU, relative light units. Data shown are m ± SEM, *n *= 3.

The viral specificity of Ank^GAG^1D4 was evaluated on HIV-Luc and Moloney murine leukemia virus (MLV)-Luc vectors, which express the luciferase-encoding reporter gene. HIV-Luc was produced in 293T cells, and MLV-Luc was produced in the GP2-293-Luc packaging cells, which stably express the MLV *gag-pol *gene products and a packageable, luciferase-encoding MLV RNA transcript [38]. HIV-Luc and MLV-Luc producer cells were transfected by the different pCEP4-ankyrin plasmids, and the extracellular media were collected at 72h posttransfection and assayed for vector yields. Vector titers were determined by measuring the luciferase activity in HIV-Luc- and MLV-Luc-infected 293T cells [39]. The expression of Myr+Ank^GAG^1D4 and Myr0Ank^GAG^1D4 in vector producer cells resulted in a 20-fold lower production of HIV-Luc (Figure 12A), while MLV-Luc production was decreased by 6- to 8-fold (Figure 12B). These results suggested a significant degree of Ank^GAG^1D4 cross-reactivity between HIV-1 and MLV, two evolutionarily divergent retroviruses. Of note, the expression of non-relevant ankyrins Myr+Ank^A3^2D3 and Myr0Ank^A3^2D3 decreased both HIV-Luc and MLV-Luc yields by a factor of 2, indicative of a basal interference level for ankyrins (Figure 12A, B).

## Discussion

Although HAART can significantly reduce HIV-1 replication and prolong the life of HIV-infected individuals [1,10], the treatment is lifelong, causes a variety of side-effects with cumulative toxicities, and is responsible for the development of resistant viral strains [40]. Moreover, host genome-integrated proviral DNA persists in latent tissue reservoirs in HIV-1-infected individuals [1,10]. All these disadvantages have led many laboratories to consider the use of anti-HIV gene therapy, either as a stand-alone approach, or as an adjuvant to pharmacological regimens [10,41]. Gene therapy offers the potential of preventing progressive HIV infection by a sustained interference with the intracellular cascade that leads to virus replication. The aim of the present study was to investigate a novel class of intracellular protein-based antiviral agents, which would interact with viral proteins at critical steps of the virus life cycle and act as intracellular inhibitors of viral replication. One particular HIV-1 protein target which still lacks specific inhibitor(s) at the stage of clinical development is the Gag polyprotein precursor Pr55Gag and the late step of viral assembly [41-49]. The viral protein that we used as the target in this study was a truncated form of Gag precursor, consisting of the MAp17 and CAp24 domains (MA-CA), with ankyrin-derived repeat proteins acting as intracellular interactors and potential blockers.

Ankyrin-repeat proteins or DARPins represent potential candidates of anti-HIV-1 molecules, since they are considered as the best alternative to intracellular antibodies, single chain antibodies or intrabodies, in terms of binding affinity, specificity and stability [12-14,23,26,27,30]. The molecular architecture of ankyrin-derived repeat proteins consists of a common structural framework made of a theoretically unlimited number of helix-turn-helix repeat motifs or modules, arranged in a parallel orientation. The same positions in consecutive repeats can accommodate variable amino acid residues, selected randomly, with the exclusion of cysteine, glycine and proline [23]. All variable amino acid side chains are oriented on the same side of the helices (refer to Figure 2B), and are presented on a large solvent-accessible, hyper-variable surface which is well adapted to bind large surfaces on desired targets [22]. More importantly, the correct folding and stability of these molecules do not depend on disulfide bridge formation and can therefore occur in all environments, including extracellular and intracellular milieu, which is a major advantage over antibodies [26,27,30]. The number of modules is theoretically unlimited, and highly variable, in natural ankyrins, but the number of ankyrin modules in selected binders is generally restricted to two or three, although longer proteins up to fifteen modules are also present in libraries [23]. This is not due to a better functional adaptation of shorter ankyrins, but likely to the proportion of coding sequences in short versus longer proteins. Long ankyrin constructs are more likely to incorporate at least one frameshift mutation; thus the useful diversity of longer sequences is limited. Nevertheless, arrangements of two to three modules have enough surface and variability to generate binders with high affinity and specificity.

The construction of a DARPin library with random amino acids at predetermined, surface exposed positions in the conserved alpha-helical module of ankyrin required the use of oligonucleotides synthesized from trinucleotide synthons. This constituted the major obstacle in the construction of such libraries, since this technology is not commonly accessible. To overcome this inconvenience, we developed an alternative strategy based on a mixture of partially degenerated oligonucleotides and generated a phage-displayed ankyrin-repeat library with a reasonable degree of diversity. What is referred to as 'useful diversity' of the ankyrin library generated in this study was estimated to be 6 × 10^7 ^independent coding sequences.

By screening our phage-displayed library on the MA-CA domains of HIV-1 Gag used as the bait, we isolated Ank^GAG^1D4, a Gag-specific, trimodular ankyrin with an apparent molecular mass of 16.5 kDa. The potential capacity of Ank^GAG^1D4 to interfere with HIV-1 replication was evaluated in SupT1 cells expressing the N-myristoylated (SupT1/Myr+Ank^GAG^1D4) and the non-N-myristoylated (SupT1/Myr0Ank^GAG^1D4) versions of Ank^GAG^1D4, respectively. We observed a lower permissiveness to HIV-1 for both cell lines, with a significant reduction of the HIV-1 progeny released in the culture medium, compared to control cells expressing Gag-irrelevant ankyrins or no exogenous ankyrin. The Ank^GAG^1D4 anti-HIV-1 effect was found to occur at the post-integration phase of the virus life cycle, a result consistent with Gag, the viral structural protein being the target of Ank^GAG^1D4. The results obtained with the MLV-Luc vector indicated that Ank^GAG^1D4 could have antiviral effect on phylogenetically distant retroviruses. Interestingly, the interferon-induced cellular protein HERC5, which acts as a host restriction factor of HIV-1 infection, has been found to block both HIV-1 and MLV Gag particle assembly with a similar efficiency [50]. The cross-reactivity observed between HIV-1 and MLV implied that Ank^GAG^1D4, which was selected on HIV-1 Gag, recognized a conformational structure or/and motif conserved among retroviral Gag prolyproteins.

The mechanism of Ank^GAG^1D4 activity was further dissected in HIV-1-infected SupT1 cells. No premature cleavage or sequestration of the Pr55Gag precursor in a cellular compartment was observed, but a lower Gag content was found in both Myr+Ank^GAG^1D4- and Myr0Ank^GAG^1D4-expressing cells, compared to control cells. Image analysis of HIV-1-infected, ankyrin-expressing SupT1 cells suggested that the non-N-myristoylated Myr0Ank^GAG^1D4 bound to nascent or newly synthesized Gag polyprotein within the cytoplasm, and that the Myr0Ank^GAG^1D4-Gag complex was addressed to the plasma membrane via the N-myristoylation signal carried by Gag. N-myristoylated Myr+Ank^GAG^1D4, however, was genetically designed for plasma membrane targeting. In both cases, the formation of Ank^GAG^1D4-Gag protein complexes likely resulted in the depletion of Gag from the pool of molecules available for virus assembly. Although these results suggested that the specific interaction of Ank^GAG^1D4 with the CA NTD negatively interfered with the Gag assembly and budding pathway at the plasma membrane, some interference with the interaction of Gag with the viral genomic RNA could not be excluded. This early interaction has been shown to occur at perinuclear/centromal sites [51] and could be the target of the cytoplasmic Myr0Ank^GAG^1D4.

The molecular and cellular mechanism of the Ank^GAG^1D4 antiviral effect might also involve the plasma membrane anchoring of Gag via its N-myristoylated signal. Several reports have shown a link between membrane anchoring of the Pr55Gag precursor and its translation. N-myristoylated Pr55Gag protein regulates its own translation *in vitro *in the presence of plasma membrane-containing fraction [52,53]. In the present study, SupT1/Myr+Ank^GAG^1D4 cells showed a lower permissiveness to HIV-1 infection, compared to SupT1/Myr0Ank^GAG^1D4, suggesting that the addressing of Ank^GAG^1D4 to the plasma membrane compartment via a N-myristoylated signal significantly increased its efficiency as antiviral agent. Furthermore, the plasma membrane-targeted, Gag-irrelevant ankyrin Myr+Ank^A3^2D3 showed some negative interference with HIV-1 replication. It might be therefore hypothesized that Myr+Ank^GAG^1D4 and Myr+Ank^A3^2D3 occupied anchoring sites in critical domains of the plasma membrane inner leaflet which were required for the insertion of Pr55Gag/genomic RNA complex and the initiation of virus assembly [54-56]. Alternatively, but not exclusively, Myr+Ank^GAG^1D4 and Myr+Ank^A3^2D3 might compete with Pr55Gag for N-myristoyl-transferases, resulting in decreased levels of N-myristoylated Pr55Gag molecules competent for plasma membrane anchoring, and viral particle formation and egress. The latter hypothesis is consistent with a previous report describing the inhibitory effect of competing unsaturated fatty acids on viral budding [57].

The binding site of Ank^GAG^1D4 was mapped to the N-terminal moiety of the CA domain, within the first 110 residues. HIV-1 CA domain is composed of two highly structured subdomains, the N-terminal subdomain (NTD, 1-145) and the C-terminal subdomain (CTD, residues 149-219), separated by a flexible hinge [58-60]. The Ank^GAG^1D4 binding site encompassed two highly accessible and functionally important regions in the CA NTD: (i) the aminoterminal ß-hairpin, and (ii) the cyclophilin-A (CypA) loop [58], which contains Proline-90 and Isoleucine-91. Pro-90 is the substrate of the CypA *cis-trans *peptidyl-prolyl isomerase, and Pro-90 and Ile-91 are two residues essential for virion incorporation of CypA, an HIV-1 infectivity factor [61-63]. Therefore, besides its effect on virus assembly, Ank^GAG^1D4 could also decrease the infectivity of HIV-1 virions, via a blockage of the CypA encapsidation.

Retroviral Gag and GagPol polyproteins are incorporated into immature virus particles through Gag-Gag and Gag-GagPol interactions. In this assembly pathway, Gag dimerization, mediated by Gag-RNA interaction, represents a critical step [54-56,64-68]. Crystal analysis of HIV-1 CA has shown that the CTD is involved in the formation of CA dimers [58-60,69,70]. If the direct interaction of Ank^GAG^1D4 with the CA NTD negatively interfered with the Gag multimerization process, this would occur via the NTD-NTD hexamerization interface, or the NTD-CTD interface, and not the CTD-CTD dimerization interface [58]. This differed from other peptide inhibitors of HIV-1 Gag assembly which have been shown to target the CTD-CTD interface and block the CA dimerization [44,46,60]. Analysis of the H_6_MA-CA/Ank^GAG^1D4 complex suggested a stoichiometry of 1:1 for the pair of reagents, and a moderate affinity of Ank^GAG^1D4 for its H_6_MA-CA target *in vitro*. As a comparison, the dodecapeptide ITFEDLLDYYGP (abbreviated CAI, for capsid assembly inhibitor), isolated by screening of a phage-displayed peptide library on the HIV-1 CA domain, has been found to bind to CA with a *K*_d _of ~ 15 μM, and to inhibit the Gag multimerization and formation of immature virus particles at an average 50% inhibitory concentration of about 10 μM [44,46].

## Conclusions

The present study demonstrated the potential of ankyrin-repeat proteins as a novel class of intracellular antivirals. The data obtained with ankyrin Ank^GAG^1D4 showed that a significant antiviral effect could be obtained with an ankyrin molecule targeted to a structural protein of the HIV-1 virion, which was the CA domain of the Gag precursor. The Ank^GAG^1D4 molecule therefore represents an attractive platform for the design of more efficient ankyrin-based intracellular inhibitors of HIV-1 which would negatively interfere with the virus assembly and egress pathway. More generally, the antiviral activity shown by Ank^GAG^1D4 should contribute to promote the use of ankyrin-repeat proteins as intracellular therapeutic agents against a variety of pathogens.

## Methods

### Cells

Human embryonic kidney cells HEK-293T cells were obtained from the American Type Culture Collection (ATCC, Manassas, VA), and maintained as monolayers in Dulbecco's modified Eagle's medium (DMEM; Invitrogen) supplemented with 10% fetal bovine serum (FBS; Invitrogen), penicillin (100 U/mL), and streptomycin (100 mg/mL) at 37°C, 5% CO2. GP2-293 and GP2-293-Luc packaging cells stably expressed the Moloney murine leukemia virus (MLV) *gag-pol *gene products, and GP2-293-Luc contained an additional packageable, luciferase-encoding viral RNA transcript [38] expressed from the luciferase reporter vector pLLRN (BD Biosciences Clontech). *Spodoptera frugiperda *(Sf9) cells were maintained as monolayers at 28°C in Grace's insect medium supplemented with 10% fetal bovine serum (FBS) and antibiotics (Invitrogen). They were infected with recombinant baculovirus at a multiplicity of infection (MOI) ranging from 2 to 10 PFU/cell, as previously described [71-74]. SupT1 cell lines stably expressing the ankyrin-repeat proteins were generated using the pCEP4-based vector (Invitrogen). Transfected SupT1 cells were maintained in complete RPMI containing hygromycin B (400 μg/mL).

### Plasmids and vectors

Plasmid pQE-30 (Qiagen) was used for production of 6xHis-tagged recombinant proteins in bacterial cells. Plasmid pNL4-3, obtained from the NIH AIDS Research and Reference Reagent Program (Division of AIDS, NIAID, NIH), was used as the template for isolation of the DNA fragments encoding the wild-type HIV-1 MA-CA and CA domains, and insertion into the pBlueBac4.5 plasmid (Invitrogen). The pBlueBac4.5 transfer vector was recombined with the genome of *Autographa californica *multiple nucleopolyhedrosis virus (AcMNPV), to generate recombinant baculoviruses AcMNPV-H_6_MA-CA and AcMNPV-H_6_CA were used to produce the H_6_MA-CA and H_6_CA recombinant proteins. The pCEP4 vector (Invitrogen) was used for constitutive, episomal expression of designed ankyrins from the CMV promoter in SupT1 cells. VSV-G-pseudotyped HIV-1-luciferase vector was recovered from the culture supernatant of 293T cells cotransfected with equal doses of pCEP4-ankyrin, phCMV-G and pNL4-3Luc(R-E-) plasmids (3 μg of each plasmid per 10^6 ^cell aliquots), as previously described [39]. VSV-G-pseudotyped MLV-luciferase vector was recovered from the culture supernatant of GP2-293-Luc cells cotransfected with equal doses (3 μg/10^6 ^cells) of pCEP4-ankyrin and phCMV-G. Cell culture supernatants containing the VSV-G-pseudotyped HIV-1 or MLV vectors (abbreviated HIV-Luc and MLV-Luc, respectively) were harvested at 72 h posttransfection, aliquoted and used for infection of 293T cells. HIV-Luc or MLV-Luc vector titers were determined at 24 h pi by luciferase assay of 293T cell lysates [39].

### Construction of ankyrin-repeat protein library

The artificial ankyrin library was constructed using a combined phage display/expression vector based on pHDiExDsbA-Ank15 [75]. This vector was used for low level expression of DARPins fused to the M13 g3p truncated protein in phage display experiments. Since this construction had also a T7 promoter and a suppressible stop codon between the DARPins and g3p coding sequences, it could also be used for periplasmic expression of nonfused DARPins in non supE strains of *E. coli *expressing T7 polymerase. DARPins are extremely stable proteins and are efficiently translocated to periplasmic space only provided that they are fused to the SRP export sequence [76]. For cytoplasmic expression, the sequence encoding soluble ankyrin proteins were inserted into the pQE-30 expression vector (Qiagen), using M15 (pREP4) strain (Qiagen) for expression. Bacterial cells XL-1 Blue MRF' (Stratagene) were used as host cells for the generation of the library and propagation of phages displaying the artificial ankyrin-repeat protein library.

The generation of our artificial ankyrin-repeat proteins library was based on that of DARPins library previously described [23], with the following modifications. A limited number of changes were introduced in the ankyrin-repeat consensus sequence in order to create a type II non-palyndromic restriction site *Bsm *BI within the ankyrin module. This site was used to produce ankyrin module-encoding microgenes from circularly amplified products and for their subsequent directional polymerization, in order to create the library. The *Bsm *BI recognition site was introduced by replacing glutamate-21 by arginine (E21R substitution) and valine-22 by leucine (V22L), using the appropriate nucleotide changes. In order to minimize possible charge repulsion involving the newly introduced R21, a compensatory change was made, consisting of a K-to-E mutation at position 25, which introduced a negative charge in the consecutive turn of the same alpha helix. These modifications were not expected to interfere with the folding or stability of the ankyrin module, since these types of amino acid residues are commonly found at equivalent positions of natural protein with ankyrin repeats. Furthermore, the changes that we created were located on the face of the protein opposite to the binding surface, and therefore should not interfere with the potential binding activity of the artificial ankyrins.

Further changes with respect to previously described libraries were introduced in the design of this library. The side chain of residue located at position 10 (helix-1) was oriented toward the binding surface and was therefore partially randomized, while position 26 and 33, not directly located within the binding surface, were kept constant. In DARPins libraries previously described, the modification of each variable amino acids of the ankyrin repeats were essentially performed randomly, with the exclusion of cysteine (C), glycine (G) and proline (P). This was made possible by using oligonucleotides synthesized from trinucleotide synthons. As this technology is not commonly accessible, we devised an alternative strategy based on a mixture of partially degenerated oligonucleotides, and comprising of the following steps (Figure 1).

(i) The oligonucleotides pools were designed to exclude undesired cysteine residues and to mimic the natural residue frequency of residues at each defined position where amino acid residues could vary, i.e. position numbers 2, 3, 5, 10, 13 and 14 (Table 1). The position-specific, natural distribution of amino-acids frequencies were computed from the natural ankyrin modules collections defined in the Prosite database (PS50088). The choice for the set of partially degenerated codons was in fact a compromise, in order to minimize the numbers of codons (and therefore of oligonucleotides), while maintaining the side chains diversity close to the chemical diversity encountered in natural ankyrin-repeat proteins.

(ii) The repeat sequences were generated by using a set of oligonucleotides containing a set of partially degenerated codons. The sequence coding a single repeat was divided into four fragments (Table 2): Va (variable fragment a), Vb (variable fragment b), Vc (variable fragment c) and C1 (constant fragment). Each variable fragment was generated by mixing a pool of oligonucleotides with randomized positions encoded with different combination of partially degenerated codons (Table 1).

(iii) All synthetic fragments (Va, Vb, Vc, and C1) were hybridized with reverse oligonucleotides linkers ("bridging" fragments; Vb-rev, C2, and C3) at equal molarity by heating at 95°C for 5 min, followed by progressive refrigeration to 25°C at the rate of 0.1°C/min.

(iv) To generate the circularized template, the hybridized product was ligated by T4 DNA ligase (New England Biolabs, NEB), purified using the NucleoSpin^® ^Extract II kit (Macherey-Nagel), and used as the template for Rolling Circle Amplification (RCA) process, using the Illustra TempliPhi 100 amplification kit (GE Healthcare, Bio-Sciences).

(v) The polymerized product was incubated at 65°C for 15 min and subsequently treated with *Bsm*B I (NEB) at 55°C for 4 h, resulting in a mixture of mono-repeat ankyrin microgenes.

(vi) The mixture of generated fragments was subjected to a hetero-polymerization process for the generation of repeat protein library, using a procedures adapted from a previous work on a different type of repeat protein [33]. In brief, the pool of mono-repeat ankyrin microgenes were inserted into and ligated to a specially designed "acceptor" vector containing the N- and C-cap of DARPins (Figure 1). This vector was first cleaved with *Bsm*B I and *Kpn *I (Fermentas) to generate the cohesive ends compatible with ankyrin repeats microgenes. The *Kpn *I cleavage, although not strictly necessary, was used to minimize the vector recircularization which would compete with ankyrin-repeat polymerisation. Once ankyrin repeats were ligated with N-Cap, vector was cleaved with *Bsp*M I (NEB) and recircularised by intramolecular ligation. This resulted in the elimination of the Rep cloning sites regions and its replacement by a variable number of ankyrin repeats between the N- and C-caps. The ligation product was transfected into electrocompetent XL-1 Blue cells. Transformed cells were selected on LB agar containing ampicillin (100 μg/mL). The number of ankyrin repeats was determined by gel electrophoresis, after digestion of the vector pool with *Not *I (NEB) and *Hind *III (NEB). The quality of the ankyrin library, based on the proportion of readthrough clones, was evaluated by CoFi blot analysis as previously described [33].

### Construction of expression vectors

*(i) Baculoviral vectors (AcMNPV)*. The baculovirus transfer vector encoding His-tagged MA-CA domains of Gag (H_6_MA-CA) was generated as described elsewhere [77]. For production of recombinant His-tagged CAp24 domain of Gag (H_6_CA), the gene encoding H_6_CA was amplified from the parental vector pNL4-3 by standard PCR protocol using pair of primers: FWD-p24 *Nhe *I, 5'-GAGGAGGAGGTGCTATAGTGCAGAACCTCCAG-3' and REV-p24 *Kpn *I, 5'-GAGGAGGAGCTGGTACCTTACAAAACTCTTGCTTTATGGCC-3'. The PCR fragment was treated with *Nhe *I and *Kpn *I and subsequently cloned into the pBlueBac4.5 transfer vector, resulting in plasmid pBlueBac-H_6_CA.

*(ii) Bacterial cell vectors (pQE-30)*. Ankyrin genes encoding H_6_MA-CA or αRep-A3-binder (αRep- previously described as αRep-n4-a (pdb-code 3LTJ; [33]) were inserted into the pQE-30 ankyrin acceptor vector, designed for soluble protein production. The acceptor vector was constructed by inserting the hybridization product of two synthetic oligonucleotides, pQE-Ank-Adapt-Fw (5'-GATCCGCGGCCGCAAACGCGTAAA-3') and pQE-Ank-Adapt-Re (5'-AGCTTTTACGCGTTTGCGGCCGCG-3'), into the *Bam *HI and *Hind *III sites of the pQE-30 vector, resulting in the insertion of a *Not *I restriction site into pQE-30. Phagemid pHDiExDsbA was treated with *Not *I and *Hind *III, and the resulting *Not *I-*Hind *III fragment was cloned into the same sites of the pQE-30 acceptor vector. The resulting pQE-30 vector contained the gene coding for Gag-binding or αRep-A3-binding ankyrin. All vector constructs were transfected into *E.coli *M15[pREP4] (Qiagen).

*(iii) Mammalian cell vectors (pCEP4)*. Two versions of ankyrin-coding vectors, pCEP4-Myr^+^Ank-GFP and pCEP4-Myr0Ank-GFP, were constructed. The N-myristoylated ankyrin-GFP fusion protein expressed by pCEP4-Myr^+^Ank^GAG^1D4-GFP was designed to be directed to the plasma membrane, whereas the non-N-myristoylated ankyrin-GFP fusion protein expressed by pCEP4-Myr0Ank^GAG^1D4-GFP was designed to localize in the cytoplasm. The DNA encoding the Gag-binders Ank^GAG^1D4 and control Ank^A3^2D3 were amplified from their respective pHDiExDsbA-encoding plasmids using two sets of primer with or without the N-myristoylation signal at the 5'end. The gene encoding the green fluorescent protein (GFP) was amplified from pTriEx-GFP [78], using primers of which sequence will be communicated upon request. PCR products encoding Ank^GAG^1D4 or Ank^A3^2D3 fused to GFP were recombined by overlapping PCR. The PCR products of the second round were treated with *Kpn *I and *Xho *I (Fermentas) and cloned into corresponding sites of the pCEP4 vector. The sequence of Ank^GAG^1D4-GFP and Ank^A3^2D3-GFP, as well as all our other constructs, was verified by standard DNA sequencing.

### Production of recombinant H_6_MA-CA and H_6_CA proteins in baculovirus-infected cells

Sf9 cells were cotransfected with 10 μg each of pBlueBac4.5-H_6_MA-CA (or pBlueBac4.5-H_6_CA) and Bac-N-Blue™ DNA, using Cellfectin^® ^II reagent, using the conditions recommended by the manufacturer (Invitrogen). The recombinant viruses obtained, BV-H_6_MA-CA and BV-H_6_CA, were isolated using the blue plaque selection method, and amplified. BV-H_6_MA-CA- and BV-H_6_CA-infected Sf9 cells were harvested at 48 h postinfection (pi), lysed by freezing and thawing. The cell lysates were clarified by centrifugation at 15, 000 × g for 30 min at 4°C. The presence of recombinant Gag proteins was detected by SDS-PAGE and Western blotting. The nitrocellulose membranes (GE Healthcare Bio-Sciences) were incubated with blocking solution (5% skimmed milk in TBS) for 1 h at RT, and Gag proteins detected using monoclonal anti-His-tag antibody (1:5, 000 dilution in the blocking solution) for 1 h at RT with slow rocking. After washing with TBST (TBS containing 0.05% Tween 20), membranes were incubated with HRP-conjugated goat anti-mouse Ig (1:8, 000 dilution in blocking solution) for 1 h at RT. After two extra washing steps, the Gag proteins were visualized using TMB membrane peroxidase substrate (KPL). His-tagged Gag proteins were purified from clarified Sf9 cell lysates by affinity chromatography on HisTrap column, using ÄKTA prime™ plus (GE Healthcare Bio-Sciences). Protein concentration was determined using the Bradford protein assay (Thermo Fisher Scientific Inc.). Purity of His-tagged Gag proteins was assessed by SDS-PAGE analysis in 15% acrylamide gel and Coomassie blue staining [77].

### Phage selection

Microtiter plate (NUNC) was coated with 100 μl H_6_MA-CA protein (or αRep-A3 protein) solution at 20 μg/mL in sterile PBS, overnight at 4°C. Purified αRep-A3 protein, produced as described [33], was used as a control for evaluating the quality of our artificial ankyrin library against a properly folded protein target. Plates were washed four times with sterile-filtered TBST. Non-specific binding was prevented by blocking with sterile-filtered blocking buffer (2% BSA in TBST; 200 μl per well) for 1 h at RT with shaking at 150 rpm on an Eppendorf Thermomixer^® ^(Eppendorf). After a washing step with TBST, 100 μl of phage suspension, corresponding to 10^11 ^particles, was added per well. After 1 h incubation at RT with shaking, plates were washed 20 times with TBST and 10 times with TBS. Substrate-bound phages were eluted by postincubation with 100 μl of 0.1 M glycine solution at pH 2.5, for 10 min at RT with shaking, followed with pH neutralization using 12.5 μl 1 M Tris-HCl buffer, pH 8. The eluted phages were mixed with 5 ml of XL-1 Blue cell suspension (OD_600 _0.6-0.8), and the mixture incubated for 30 min at 37°C. Bacterial cells were centrifuged at 1, 200 × *g *for 10 min at 25°C, pellet resuspended in 1 ml 2X YT broth and plated on LB agar containing ampicillin (100 μg/mL). Bacterial colonies were pooled, and used for phage preparation to perform the next round of phage selection. Individually picked, single colonies of the second and third rounds of selection were screened by phage ELISA.

### Expression and purification of soluble ankyrins with Gag-binding activity

M15[pREP4] bacterial cells harboring the pQE30-ankyrin plasmid were grown in 500 ml LB broth supplemented with ampicillin (100 μg/mL), kanamycin (25 μg/mL), and 1% (w/v) glucose, at 37°C with shaking until OD_600 _reached 0.8. Protein expression was induced by addition of 1 mM IPTG, and maintained in culture for 4 hr at 30°C with shaking. Bacteria were pelleted by centrifugation at 1, 200 × *g *for 30 min at 4°C. Pellets were resuspended in lysis buffer and subjected to three cycles of freezing and thawing. Lysis buffer consisted of TBS buffer, pH 7.4, containing 1 μg/mL lysozyme and a cocktail of protease inhibitors (Roche Diagnostics GmbH). Bacterial cell lysates were clarified by centrifugation at 15, 000 × *g *for 30 min at 4°C. The soluble form of Gag-interacting ankyrins was purified from the clarified bacterial lysates by a two-step procedure comprising of affinity chromatography on HisTrap column followed by gel filtration on Sephadex G-75 (GE Healthcare Bio-Sciences). Proteins were analyzed by SDS-PAGE and Coomassie blue staining, or SDS-PAGE and Western blotting, as detailed below.

### Biotinylation of soluble ankyrins

Purified Gag-binding ankyrins were chemically biotinylated using the EZ-Link Sulfo-NHS-LC-Biotin kit (ThermoScientific, Rockford, IL). In brief, a solution of purified protein at 100 μM was mixed with a 5-fold molar excess of Sulfo-NHS-Biotin solution in a final volume of 2 ml, and incubated at 25°C for 1 h. Excess reagents and by-products were removed by applying the mixture to a pre-equilibrated Zeba™ Desalt Spin column (ThermoScientific). The column was centrifuged at 1, 000 × g for 2 min, and the biotinylated proteins were recovered in the flow-through fraction. The concentration of biotinylated proteins was determined using the NanoDrop 2000 system (ThermoScientific). The biotinylation efficiency of proteins was qualitatively evaluated using dot-blot analysis. 10 μmol biotinylated proteins was spotted on nitrocellulose membrane, membrane blocked with blocking buffer (5% BSA in TBS), and biotin groups revealed by extravidin-HRP (Sigma) used at dilution 1:5, 000 in blocking buffer (1 h at RT with shaking) and BM Blue POD Substrate (Roche Diagnostics GmbH).

### Assessment of ankyrin reactivity towards HIV-1 MA-CA polyprotein

*(i) Competitive ELISA*. Microtiter plates were coated with 100 μl of purified H_6_MA-CA or αRep-A3 (1 μg/mL) diluted in PBS and left overnight at 4°C in a moisture chamber. The coated wells were washed four times with TBST and incubated with 200 μl blocking solution (2% BSA in TBS) for 1 h at RT. After washing, 100 μl biotinylated Gag-binding ankyrin at 10 μM, alone or mixed with an equal molar amount of competitor (non-biotinylated ankyrin or irrelevant ankyrin), was added and incubated for 1 h at RT. Plates were then washed and incubated with extravidin-HRP diluted to 1:5, 000 in blocking solution for 1 h at RT. After washing, 100 μl of TMB substrate was added, and the reaction was blocked by addition of 1 N HCl. OD was measured at 450 nm using a MTP-120 ELISA plate reader (Corona Electric, Ibaraki, Japan).

*(ii) Far Western blotting*. Lysates of Sf9 cells infected by BV-H_6_MA-CA were analyzed by SDS-PAGE and proteins transferred to polyvinylidene fluoride (PVDF) membrane (GE Healthcare Bio-Sciences). Membranes were incubated in blocking buffer (5% BSA in TBS) overnight at 4°C, then postincubated with biotinylated Gag-binding ankyrins at 1 μM for 1 h at RT with gentle rocking. Substrate-bound biotinylated-ankyrins were detected by reaction with extravidin-HRP (diluted to 1:10, 000 in blocking buffer) and TMB membrane peroxidase substrate (KPL).

### Mapping of ankyrin binding site on HIV-1 Gag precursor

*(i) Specificity assay*. The specificity of the Gag-binding ankyrins towards the CA domain was performed by indirect ELISA. Lysates of BV-H_6_CA-infected cells were added to nickel pre-coated wells, as described elsewhere [79]. Biotinylated Gag-binding ankyrins were individually reacted with immobilized H_6_CA domain for 1 h at 37°C. The binding reaction was monitored by adding extravidin-HRP (dilution 1:5, 000) and TMB substrate. After stopping reaction with 1N HCl, the signals were measured at OD_450_, as above described.

*(ii) Mapping*. The ankyrin binding site on the CA domain was determined using Gag amber mutants (Gag*amb*) expressed as recombinant proteins in baculovirus-infected cells [36]. Lysates of Sf9 cells expressing Gag*amb*276 or Gag*amb*241 polyprotein were coated on ELISA plates and reacted with biotinylated Gag-binding ankyrins, as above.

### Microcalorimetry analysis of Gag-ankyrin binding parameters

Interaction between proteins was analyzed by isothermal titration calorimetry (ITC), using the MicroCal iTC_200 _isothermal titration microcalorimeter (Microcal), under the conditions described in a previous study [80]. All proteins were diluted in 20 mM phosphate buffer pH7.5, 150 mM NaCl. For each injection, 2 μl of ankyrin solution was added from a computer-controlled 40-μl microsyringe at intervals of 180 s into the protein substrate solution, H_6_MA-CA or αRep-A3. A theoretical titration curve was fitted to the experimental data, as previously described [80].

### Construction of cell lines stably expressing ankyrins

Aliquots of SupT1 cells (10^6 ^cells) were electroporated with pCEP4-based vectors encoding GFP-fused ankyrins with (Myr+) or without (Myr0) the myristoylation signal, using the Nucleofector™ (Lonza, Basel, Switzerland) and the Nucleofector™ transfection reagent V (Lonza), according to protocol T-16. Transfected cells were maintained in complete RPMI containing hygromycin B (400 μg/mL). Four cell lines were generated, SupT1/Myr+Ank^GAG^1D4-GFP, SupT1/Myr0Ank^GAG^1D4-GFP, SupT1/Myr+Ank^A3^2D3-GFP and SupT1/Myr0Ank^A3^2D3-GFP, respectively. The level of expression of Ank^GAG^1D4-GFP and control Ank^A3^2D3-GFP proteins was monitored by flow cytometry of the GFP signal, and cellular localization by confocal fluorescence microscopy. For flow cytometry, cells were blocked by incubation with human AB serum on ice for 30 min. They were reacted with 50 μl of purified anti-CD4 mAb MT4-3 [81] at 20 μg/mL in 1% BSA-PBS-NaN_3 _on ice for 30 min. At the end of the incubation time, the cells were washed three times with PBS, and incubated with 25 μl PE-conjugated rabbit anti-mouse F(ab')2 (DAKO) on ice for 30 min. Cells were washed, fixed in 1% paraformaldehyde in PBS, and analyzed by flow cytometry.

### HIV-1 challenge

To evaluate the effect of Gag-binding ankyrins on the HIV-1 life cycle, SupT1 cells stably expressing the Myr^+ ^or Myr0 version of the best Gag binder Ank^GAG^1D4 and irrelevant control Ank^A3^2D3, were challenged with HIV-1 virions (NL4-3 strain). Triplicate samples of SupT1/Myr+Ank^GAG^1D4, SupT1/Myr0Ank^GAG^1D4, SupT1/Myr+Ank^A3^2D3 and SupT1/Myr0Ank^A3^2D3 were infected at MOI 10 for 16 h at 37°C. The virus infectivity titer was determined from the genome copy number measured by quantitative RT-PCR (Roche Diagnostics). Cells were then washed three times with serum-free medium, resuspended in 3 mL of fresh medium containing 400 μg/mL hygromycin B and 10% FCS, and seeded into 6-well plates. Cells were harvested at days 5, 7, 9, 11 and 13, and culture supernatants and cell pellets were separately processed for determination of virus progeny yields and viral integration.

### HIV-1 production assay

The yields of extracellular virus were evaluated in triplicate samples of culture supernatants of day 11 pi, using a CAp24 ELISA kit (Genscreen ULTRA HIV Ag-Ab, BioRad). Day-11 samples were also assayed for viral genome copy numbers, using COBAS^® ^AmpliPrep/COBAS TaqMan HIV-1 Test (Roche Diagnostics GmbH). Extracellular budding of virions was also monitored by syncytium formation observed in day-11 samples under an inverted microscope (Olympus).

### Gag protein assays

Membrane-bound and particulate form of Gag proteins were determined in HIV-1-infected SupT1 cells subjected to cell fractionation. Triplicate cell samples of days 9 and 11 pi were lysed and extracted using the FractionPREP™ Cell Fractionation System (BioVision, Mountain View, CA), following the manufacturer's instructions. The membrane fraction thus isolated was assayed for HIV-1 Gag protein content, using the CAp24 ELISA kit mentioned above, or SDS-polyacrylamide gel electrophoresis (SDS-PAGE) and Western blotting. Proteins were denatured by heating to 100°C for 2 min in SDS-ß-mercaptoethanol-containing sample buffer, electrophoresed in SDS-containing 15%-polyacrylamide gel [77], and then electrically transferred to a polyvinylidene-fluoride (PVDF) membrane. PVDF membranes were blocked with 5% skimmed milk in PBS containing 0.5% Triton X-100, then probed with anti-CAp24 monoclonal antibody (mAb) G18, or anti-MAp17 mAb M48. Both G18 and M48 mAbs were laboratory-made (W. Kasinrerk; unpublished). Blots were developed using HRP-conjugated goat anti-mouse IgG antibody and TMB membrane peroxidase substrate. Extracellular virus-like particles (VLP) released by MLV Gag-Pol-expressing GP2-293 cells were recovered by ultracentrifugation of the cell culture medium [71,82], and VLP production estimated by SDS-PAGE of VLP pellets and Western blot analysis using rabbit polyclonal antibody to MLV-GagCAp30 protein (antibodies-online Inc., Atlanta, GA). Intracellular content of MLV Gag proteins was analyzed in the same manner, using the whole cell lysate.

#### HIV-1 integration assay

The number of viral genome copies integrated into the host DNA of control SupT1 or SupT1 expressing Gag-specific (Ank^GAG^1D4) or irrelevant ankyrin (Ank^A3^2D3) was determined using a conventional *Alu-gag *qPCR assay [83,84] with some modifications. The first-round of PCR was performed on cellular DNA, extracted using the High Pure PCR Template Preparation Kit (Roche, Mannheim, Germany). The sequences of the first round amplification primers were: *Alu *forward, 5'-GCC TCC CAA AGT GCT GGG ATT ACA G-3' [84], and HIV-1 *gag *reverse, 5'- GTT CCT GCT ATG TCA CTT CC -3' [83]. The first round reactions were carried out in a volume of 25 μl containing 2.5× master mix (5 PRIME, Gaithersburg, MD), using a standard protocol. The second-round of real-time quantitative PCR of RU5 was performed using 10 μl of diluted (1:8) first-round amplicons. The sequences of primers were: R_FWD, 5'-TTA AGC CTC AAT AAA GCT TGC C-3'; and U5 _REV, 5'-GTT CGG GCG CCA CTG CTA GA-3', and the sequence of RU5 molecular beacon probe was 5'-FAM-CCA GAG TCA CAC AAC AGA CGG GCA CA-BBQ-3' [85]. The reactions were carried out in a final volume of 25 μl containing 2× DyNAmo probe qPCR master mix (Finnzymes, Espoo, Finland), 400 nM RU5 (R_FWD) primer, 400 nM RU5 (U5 _REV) primer, and 140 nM RU5 molecular beacon probe. The reactions were performed in a MJ Mini Thermal Cycler and MiniOpticon Real-Time PCR System (BioRad) with the following thermal program: 20-sec hot start at 95°C followed by 50 cycles of denaturation at 95°C for 3 sec and annealing and extension at 63°C for 30 sec. A primer-probe set, designed to quantify the copy number of the cellular gene glyceraldehyde 3-phosphate dehydrogenase (*GAPDH*), was used to quantify the amount of DNA in each qPCR assay. The *GAPDH *primer sequences were as follows: GAPDH_FWD, 5'-GAA GGT GAA GGT CGG AGT C-3'; and *GAPDH*_REV, 5'-GAA GAT GGT GAT GGG ATT TC-3'. The GAPDHTM molecular beacon probe was designed to contain the following sequence: 5'-FAM-CAA GCT TCC CGT TCT CAG CCT-BBQ-3'. The reactions were carried out as described above. Results were expressed as *C*ts, i.e. the number of cycles (*C*ts) required for the fluorescence signal to cross the threshold value (cycle threshold). Control experiments for the inhibition of provirus integration in HIV-1-infected cells were carried out as follows. The HIV-1 integrase inhibitor Raltegravir™ (RAL; Merck Sharp & Dohme) was added at 0, 1, 10, and 100 nM, respectively, to SupT1 cell culture medium 24 h prior to HIV-1 infection [37]. SupT1 cells were then infected with HIV-1_NL4-3 _at MOI 10, and virus inoculum removed after 16 h. Cells were washed three times with prewarmed, serum-free medium and resuspended in growth medium containing RAL at the above-mentioned concentrations. Cells were divided (1:2) every second day to maintain a cell density of approximately 10^6 ^cells per mL, and harvested at day-7 pi for *Alu-gag *and *GAPDH *qPCR assays. Cell viability in each sample was assayed using PrestoBlue Cell Viability Reagent (Invitrogen).

#### HIV-1 *gag *gene sequencing

Mock-infected or HIV-1-infected SupT1 cells (MOI 10) expressing Myr+Ank^GAG^1D4 or Myr+Ank^A3^2D3, were harvested at day-13 pi. A four-step protocol was then applied. (i) Total viral RNA was isolated, using the High Pure Viral RNA kit (Roche Applied Science, Roche, Mannheim, Germany). (ii) The viral RNA thus obtained was reverse transcribed into cDNA, using the Transcriptor High Fidelity cDNA Synthesis Kit with anchored-oligo(dT)_18 _primer (Roche). (iii) The single-stranded cDNA was then amplified, using a proof-reading PCR protocol (Phusion™ High-Fidelity DNA Polymerase; Finnzymes, Espoo, Finland) and the following pair of p24-specific gene primers: FWD_p24 *Nhe *I, 5'-GAGGAGGAGGTGCTAGCCCTATAGTGCAGAACCTCCAG-3' and REV_p24 *Kpn *I, 5'-GAGGAGGAGCTGGTACCTTACAAAACTCTTGCTTTATGGCC-3'. (iv) The PCR products were purified using the GeneJET™ PCR purification kit (Fermentas International), and sequenced using standard DNA sequencing method (1st BASE Pte Ltd, Singapore).

#### Confocal microscopy

Aliquots of HIV-1-infected SupT1 cells (1 × 10^6 ^cells, MOI 10) were harvested on day 11 pi, washed with PBS, fixed in 4% formaldehyde in PBS, and permeabilized with 0.2% Triton X-100. After blocking with 10% human AB serum for 30 min at room temperature, cells were incubated with G18 anti-CAp24 mAb at 37°C for 1 hr. After washing twice with PBS containing 1% BSA and NaN_3_, cells were incubated with PE-conjugated polyclonal rabbit anti-mouse IgG F(ab')_2 _(Dako, Denmark), and nuclei counterstained with DAPI. Images were acquired using FluoView laser scanning confocal microscope (Olympus, FV1000; Olympus Optical, Japan).

## Conflict of interests

The authors declare that they have no competing interests.

## Authors' contributions

Conceived and designed the experiments: SN AU SSH PB PM CT. Performed the experiments: SN AU MVL WK SS SSH PM. Analyzed the data: SN MVL WK SSH PM CT. Wrote the paper: SN SSH PB PM CT. All authors approved the submitted manuscript.

## Supplementary Material

Additional file 1 **Control, Raltegravir-mediated inhibition of HIV-1 integration in SupT-1 cells**. Aliquots of HIV-1-infected SupT1 cells were pretreated with Raltegravir at 0, 1, 10, and 100 nM, respectively, for 24 h prior to HIV-1 infection (MOI 10). The drug was maintained at the indicated concentrations for 7 days, and the cells harvested at day 7 pi. The level of HIV-1 integration in SupT1 cell lines was evaluated by quantitative PCR amplification of host cell DNA extracts, using primers specific to *Alu*-*gag *junctions and to cellular *GAPDH *gene as the internal control. **(A)**, *Alu-gag *qPCR obtained with the different cell samples. The qPCR assays were performed in triplicate. The colours of the curves correspond to the different Raltegravir molarities, as indicated in (B). **(B)**, Comparison of the mean *C*ts values (m ± SD) for *Alu-gag *and *GAPDH *qPCR. ND, not detectable (below the detection threshold). **(C)**, Cell viability, determined by the PrestoBlue Cell Viability Reagent, and expressed as the percentage of control, untreated cells. Click here for file
