# Supplementary figures and images for: Antiviral activity of recombinant ankyrin targeted to the capsid domain of HIV-1 Gag polyprotein
Source: Retrovirology. 2012 Feb 20;9:17. doi: 10.1186/1742-4690-9-17 (PMC3308923; doi:10.1186/1742-4690-9-17)

A

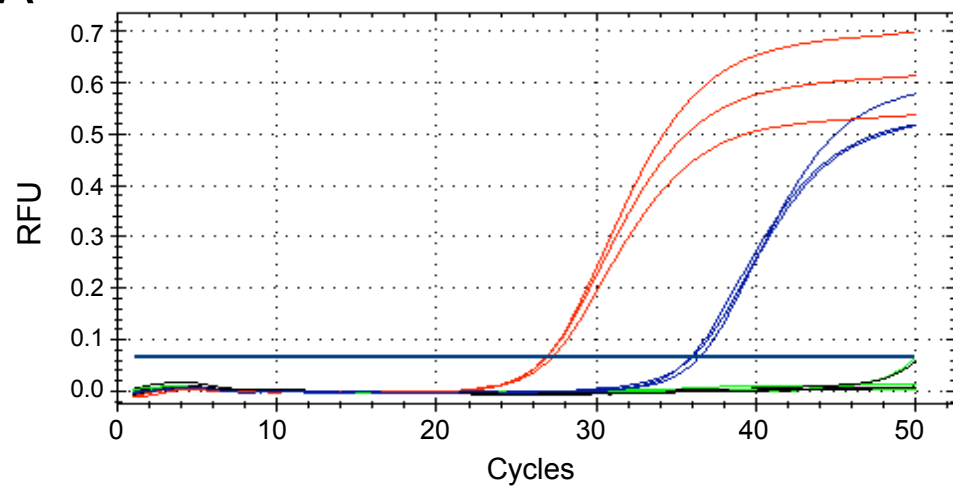

B

| Raltegravir<br>(nM) | <i>Alu-gag</i> qPCR<br>(mean Cts ± SD) |
|---------------------|----------------------------------------|
| 0                   | 26.9 ± 0.2                             |
| 1                   | 36.1 ± 0.3                             |
| 10                  | ND                                     |
| 100                 | ND                                     |

C

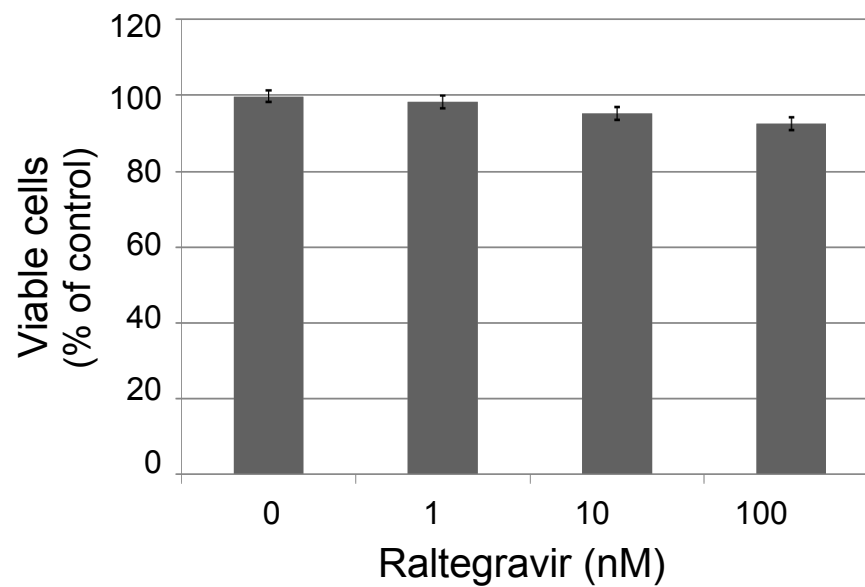

Supplement: Additional file 1 — Control, Raltegravir-mediated inhibition of HIV-1 integration in SupT-1 cells. Aliquots of HIV-1-infected SupT1 cells were pretreated with Raltegravir at 0, 1, 10, and 100 nM, respectively, for 24 h prior to HIV-1 infection (MOI 10). The drug was maintained at the indicated concentrations for 7 days, and the cells harvested at day 7 pi. The level of HIV-1 integration in SupT1 cell lines was evaluated by quantitative PCR amplification of host cell DNA extracts, using primers specific to Alu-gag junctions and to cellular GAPDH gene as the internal control. (A), Alu-gag qPCR obtained with the different cell samples. The qPCR assays were performed in triplicate. The colours of the curves correspond to the different Raltegravir molarities, as indicated in (B). (B), Comparison of the mean Cts values (m ± SD) for Alu-gag and GAPDH qPCR. ND, not detectable (below the detection threshold). (C), Cell viability, determined by the PrestoBlue Cell Viability Reagent, and expressed as the percentage of control, untreated cells. [file 1742-4690-9-17-S1.PDF]
